# Glioma stem cell signature predicts the prognosis and the response to tumor treating fields treatment

**DOI:** 10.1111/cns.13956

**Published:** 2022-09-07

**Authors:** Bo Chen, Xiaoxi Zhou, Liting Yang, Hongshu Zhou, Ming Meng, Hao Wu, Zhixiong Liu, Liyang Zhang, Chuntao Li

**Affiliations:** ^1^ Department of Neurosurgery, Xiangya Hospital Central South University Changsha China; ^2^ National Clinical Research Center for Geriatric Disorders, Xiangya Hospital Central South University Changsha China; ^3^ Hypothalamic‐Pituitary Research Center, Xiangya Hospital Central South University Changsha China; ^4^ Clinical Diagnosis and Therapy Center for Glioma, Xiangya Hospital Central South University Changsha China; ^5^ Department of Neurosurgery, The Third Xiangya Hospital Central South University Changsha China

**Keywords:** glioma stem cell, machine learning, prognostic model, tumor‐treating field

## Abstract

**Introduction:**

Glioma stem cells (GSCs) play an important role in glioma recurrence and chemo‐radiotherapy (CRT) resistance. Currently, there is a lack of efficient treatment approaches targeting GSCs. This study aimed to explore the potential personalized treatment of patients with GSC‐enriched gliomas.

**Methods:**

Single‐cell RNA sequencing (scRNA‐seq) was used to identify the GSC‐related genes. Then, machine learning methods were applied for clustering and validation. The least absolute shrinkage and selection operator (LASSO) and COX regression were used to construct the risk scores. Survival analysis was performed. Additionally, the incidence of chemo‐radiotherapy resistance, immunotherapy status, and tumor treating field (TTF) therapy response were evaluated in high‐ and low‐risk scores groups.

**Results:**

Two GSC clusters exhibited significantly different stemness indices, immune microenvironments, and genomic alterations. Based on GSC clusters, 11‐gene GSC risk scores were constructed, which exhibited a high predictive value for prognosis. In terms of therapy, patients with high GSC risk scores had a higher risk of resistance to chemotherapy. TTF therapy can comprehensively inhibit the malignant biological characteristics of the high GSC‐risk‐score gliomas.

**Conclusion:**

Our study constructed a GSC signature consisting of 11 GSC‐specific genes and identified its prognostic value in gliomas. TTF is a promising therapeutic approach for patients with GSC‐enriched glioma.

## Graphical abstract

We divided glioma patients into the high‐ and low‐glioma stem cell (GSC) groups. GSC‐enriched patients had higher chemo‐radiotherapy resistance and worse prognosis than low‐GSC patients. Tumor treating field (TTF) therapy can effectively inhibit malignant biological characteristics of GSC‐enriched gliomas, including mitosis, angiogenesis, migration and invasion potential. TTF also could break the original DNA balance in GSC‐enriched gliomas. Taken together, TTF is a promising therapeutic approach for GSC‐enriched glioma patients.

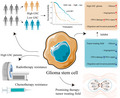

## 1 INTRODUCTION

Gliomas, derived from neuroglial stem or progenitor cells, are the most common primary malignant brain tumors, with an incidence of 6.6 per 100,000 people.^1^, ^2^ According to histological features, the World Health Organization (WHO) stratified gliomas into I–IV grades, including low‐grade glioma (LGG) of grade II–III and glioblastoma (GBM) coded grade IV. The median overall survival (OS) time of LGG is 78.1 months, while the median OS time of GBM is only 14–16 months.^3^ The current treatment regimen for glioma patients is maximal surgical resection followed by radio‐chemotherapy; however, survival remains unsatisfactory due to recurrence and drug resistance.^4^

Glioma tumor stem cells (GSCs) are a minimized subpopulation of glioma cells characterized by their stem cell properties. Self‐renewal endows GSCs with differentiated progenies that constitute the bulk of glioma mass.^5^ The Biomarkers of GSCs include CD133, CD44, CD15, and others, but are not considered precise predictors.^6^ Based on molecular signatures, GSCs can be further divided into proneural GSCs (pGSCs) and mesenchymal GSCs (mGSCs).^7^ With chemo‐radiotherapy procedures, adaptive transitions may occur between pGSCs and mGSCs.^8^ Recent studies have indicated that GSCs are partially responsible for the occurrence of glioma recurrence and chemo‐radiotherapy resistance.^9^, ^10^

Tumor treating fields (TTFs) is a novel noninvasive antitumor treatment that uses low‐intensity, intermediate frequency, and alternating electric fields.^11^ The electric fields in mitotic cells are nonuniform, whereas in static cells, it is uniform.^12^ TTFs can induce tumor cell death by interfering cell division, enhancing the ant‐itumor immune responses, promoting DNA damage, and inhibiting DNA repair.^13^ In addition, TTFs can also interfere with tumor cell functions like inhibiting migration, invasion, and angiogenesis; increasing Ca^2+^ influx; and transforming membrane permeability.^13^ When combined with conventional chemo‐radiotherapy, TTFs increase the treatment efficiency and sensitivity without elevating systemic toxicity.^14^ Recent studies presented that TTFs can induce GSC autophagy and act synergistically with immunotherapy targeting GSCs to reduce chemoresistance.^15^, ^16^ Although some studies have pointed out the potential roles of TTFs in GSCs, the in‐depth effect of TTFs on GSC‐associated glioma subtypes and therapy resistance is still discovered unsatisfactorily.

Single‐cell RNA sequencing (scRNA‐seq) provides an encouraging method to identify marker genes of specific cells. In this study, scRNA‐seq was used to identify GSC‐related marker genes. Accordingly, machine learning methods, including non‐negative matrix factorization (NMF) and random forest (RF), were used to divide and validate patient clustering by referring these genes. Subsequently, least absolute shrinkage and selection operator (LASSO) regression and Cox regression were performed to construct risk scores, using the differently expressed GSC genes between the stratified groups. Finally, we applied GSCs risk scores to predict TTFs therapeutic sensitivity comparing to the chemo‐radiotherapy resistance and immunotherapy. This study expectantly developed personalized therapeutic approaches of TTFs targeting GSCs and served as a basis for future research on GSCs in gliomas.

## 2 MATERIALS AND METHODS

### 2.1 Data source and acquisition

Single‐cell transcriptome files of four glioma samples from GSE84465 and eight glioma samples from Chinese Glioma Genome Atlas (CGGA) scRNA‐seq were downloaded from the Gene Expression Omnibus (GEO, https://www.ncbi.nlm.nih.gov/geo/) and CGGA (http://www.cgga.org.cn/index.jsp) databases, separately. The single‐cell transcriptome was used to screen for GSCs marker genes. The bulk tumor transcriptome data and clinical information of 698 and 1018 glioma samples were downloaded from The Cancer Genome Atlas (TCGA, https://portal.gdc.cancer.gov/) and CGGA databases, separately. Bulk tumor transcriptome was performed to divide subgroups based on marker genes. Of the 1728 patients enrolled in our study, GEO and TCGA data were used as the training set and CGGA data were used as the validation sets. To further investigate the genetic features of the subgroups, somatic mutations and somatic copy number alternations (CNAs), which corresponded to the patients with bulk tumor transcriptome data, were downloaded from the TCGA database. Bioinformatics analysis in our study utilized public databases, where the original studies obtained ethical approval. Informed consent was obtained from all the patients.

### 2.2 Identification of GSC marker genes by scRNA‐seq analysis

The scRNA‐seq data were processed using the R packages “Seurat” (version 4.0.6) and “harmony” (version 0.1.0).^17^ Firstly, we used “NormalizeData” to normalize the expression matrix, “FindVariableFeatures” to identify the top 2000 variably expressed genes, and “ScaleData” to exclude the effects of mitochondria and cell cycle genes. Afterward, “RunHarmony” was used to remove the batch effect among different samples. Then, the dimensionality was reduced using principal component analysis (PCA), the cells were combined together in a 2.0 resolution using the “FindClusters”, and visualized cell clustering using T‐distributed stochastic neighbor embedding (t‐SNE). Cell annotation was performed using the R packages “SingleR” and “irGSEA”. The mGSCs and pGSCs signatures were obtained from Wang's research.^7^ We extracted marker genes (P < 0.05 and log2‐fold change >1 or <−1) of cell subclusters with high GSC enrichment scores and took the intersection between the training and validating cohorts. Gene ontology (GO) and Kyoto Encyclopaedia of Genes and Genomes (KEGG) were used for functional annotation of interesting marker genes.

### 2.3 Construction and validation of GSC subtypes

Based on 146 intersected maker genes extracted from scRNA‐seq, the NMF algorithm was applied to identify robust clusters of TCGA patients.^18^ Consensus heatmaps were used to assess the optimal cluster number of 2. To validate the stability of the subtype, we trained an RF classifier in the TCGA training cohort to predict GSC glioma subtypes in the two CGGA validating cohorts. the number of trees was set to 100. The clustering performance was evaluated using the *p*‐value, precision, and *F*‐measure.

### 2.4 Stemness index in GSC subtypes

The stemness indices of TCGA glioma samples were obtained from Malta's research.^19^ To evaluate the stemness of GSC subtypes, we compared the expression levels of mRNAsi, EREG‐mRNAsi, mDNAsi, EREG‐mDNAsi, DMPsi, and ENHsi in GSC subtypes.

### 2.5 Annotation of the immune infiltrating microenvironment

The Xcell algorithm was performed to evaluate the enrichment level of 64 types of immune and stromal cells.^20^ The immune scores and stromal scores were quantified by the Estimate algorithm.^21^ The 22 types of infiltrating immunocyte fractions were calculated using the CIBERSORT algorithm after the voom normalization.^22^ In addition, the expression levels of seven types of immune checkpoints,^23^, ^24^ were compared among different GSC clusters.

### 2.6 Genomic alterations in GSC subtypes

To investigate the genetic features of the two clusters, arm‐ and focal‐level somatic CNAs were calculated using the GISTIC 2.0 analysis (https://cloud.genepattern.org/). The variant types, variant classifications, and single‐nucleotide polymorphism were compared. Significant tumor‐mutated genes (*q*‐value <0.05) across the two clusters were identified using the MutSigCV algorithm.^25^ Afterward, the interaction effect and GO functional annotation of the significantly mutated genes were further analyzed.

### 2.7 Identification of a GSC‐related signature

LASSO and univariate COX regressions were applied to identify the differentially expressed GSC genes with non‐zero penalty coefficient and prognostic significance (*p* < 0.001) between the two clusters. The GSC risk score of each patient was constructed by weighting the LASSO penalty coefficient. The median value of risk scores was set as the cutoff to divide glioma patients into low and high risk.

### 2.8 Construction and validation of a prognostic model

Univariate and multivariate Cox regression analyses were used to identify the independent prognostic factors. A nomogram model was then constructed for the survival prediction of patients with glioma. Calibration and receiver operating characteristic (ROC) curves were used to evaluate the predictive accuracy and performance of the nomogram model.

### 2.9 Evaluation of chemo‐radiotherapy effect

The R package “oncoPredict” was conducted to predict patient sensitivity to temozolomide (TMZ), and a lower IC_50_ value represented higher sensitivity.^26^ Chemo‐radiotherapy resistance was also identified by feature genes (chemoresistance: ADAM8,^27^ CASP8,^28^ FERMT3,^29^ HMGA1,^30^ ID1,^31^ ID4,^32^ IKBKE,^33^ MSI1,^34^ NAP1L1,^35^ NT5E,^36^ NUSAP1,^37^ PYCARD,^38^ RAD18,^39^ REV3L,^40^ TRIM14^41^ and TRIM24^42^; Radioresistance: RAD18,^43^ SP1,^44^ CD81,^45^ SERPINA3,^46^ ALKBH5,^47^ E2F8,^48^ CXCL1,^49^ IGF1R,^50^ CD44,^51^ RCC2,^52^ MSI1,^53^ CTSB,^54^ ITGA6,^55^ FGFR1,^56^ TAX1BP3,^57^ and BMI1^58^) and quantified by Gene Set Variation Analysis (GSVA) scores.

In addition, we also correlated the GSC risk score and TMZ sensitivity in our previously proposed glioma model integration system (patient‐derived glioma organoids and xenografts).^59^ The experimental workflow is illustrated in Figure 4A. Briefly, we isolated, cultured, and sequenced the glioma cells from patients and transplanted the cells into the cerebral organoids. Subsequently, we performed the TMZ susceptibility tests on both the patient‐derived glioma cells and cerebral glioma organoids.

### 2.10 Prediction of immunotherapy response

Neoantigens and six types of immune subtypes, obtained from Thorsson's research,^60^ were used to identify the immune landscape of gliomas. Microsatellite instability and immune checkpoints originated from the previous studies,^23^, ^24^, ^61^ were used to infer the immunotherapy response.

### 2.11 Prediction of TTF therapy sensitivity

TTF therapy sensitivity was evaluated by five malignant biological features of gliomas: mitosis, angiogenesis, DNA repair, DNA damage, migration, and invasion.^13^ Two mitosis‐related gene sets (CELL CYCLE PHASE, M PHASE OF MITOTIC CELL CYCLE) and one angiogenesis‐related gene set (ANGIOGENESIS) were downloaded from MsigDB v7.0 and quantified by GSVA scores. DNA repair capacity was evaluated by IDH and MGMT (DNA repair enzyme).^62^, ^63^ DNA damage level was inferred by major categories, including aneuploidy, CNA burden, and intratumor heterogeneity (ITH), data of which were obtained from published studies.^60^ TTF treatment targets for tumor migration and invasion were identified by the expression level of vimetin (VIM), E‐cadherin (CDH1), and fibronectin (FN1).^13^

### 2.12 TTF treatment of the study patients

Written informed consent was obtained from all patients. The study methodologies were approved by the Ethics Committee of Xiangya Hospital, Central South University (registration number: ChiCTR2100047049; Ethics approval number:202107115). Patients enrolled in the trials received TTFs (150 kHz for >18 hours/day) by ASCLU‐300 TTF, which was designed by Hao Wu et al. and has been approved by the China Institute of Food and Drug verification (Beijing, China).^64^ Safety was assessed according to CTCAE (Common Terminology Criteria for Adverse Events) Version 4.0. Magnetic resonance imaging (MRI) was performed every 3 months to evaluate the tumor response.

### 2.13 Statistical analysis

Statistical analysis was carried out using R software (version 4.1.2). Machine learning was performed by Weka software (version 3.8.4). Kaplan–Meier curves with the log‐rank test were used to compare the survival rates between the two groups. Univariate and multivariate Cox regression was applied to explore prognostic factors. Normal distribution was examined by Shapiro–Wilk normality tests. For continuous variables, the two‐tailed t‐test (normal distribution) and Mann–Whitney test (non‐normal distribution) were used to compare the two groups. For categorical variables, χ^2^ test and Fisher's exact test were used to compare the two groups. Spearman's correlation was performed to calculate correlation coefficients between continuous variables. Data were visualized using the R package “ggplot2”. Heatmap was generated based on the R package “pheatmap”. Survivorship curves were drawn with the R package “survminer”. NS indicated not statistically significant; *p < 0.05; **p < 0.01; ***p < 0.001. *p* < 0.05 was considered statistically significant.

## 3 RESULTS

### 3.1 Identification of GSC marker gene expression profiles

**FIGURE 1:**
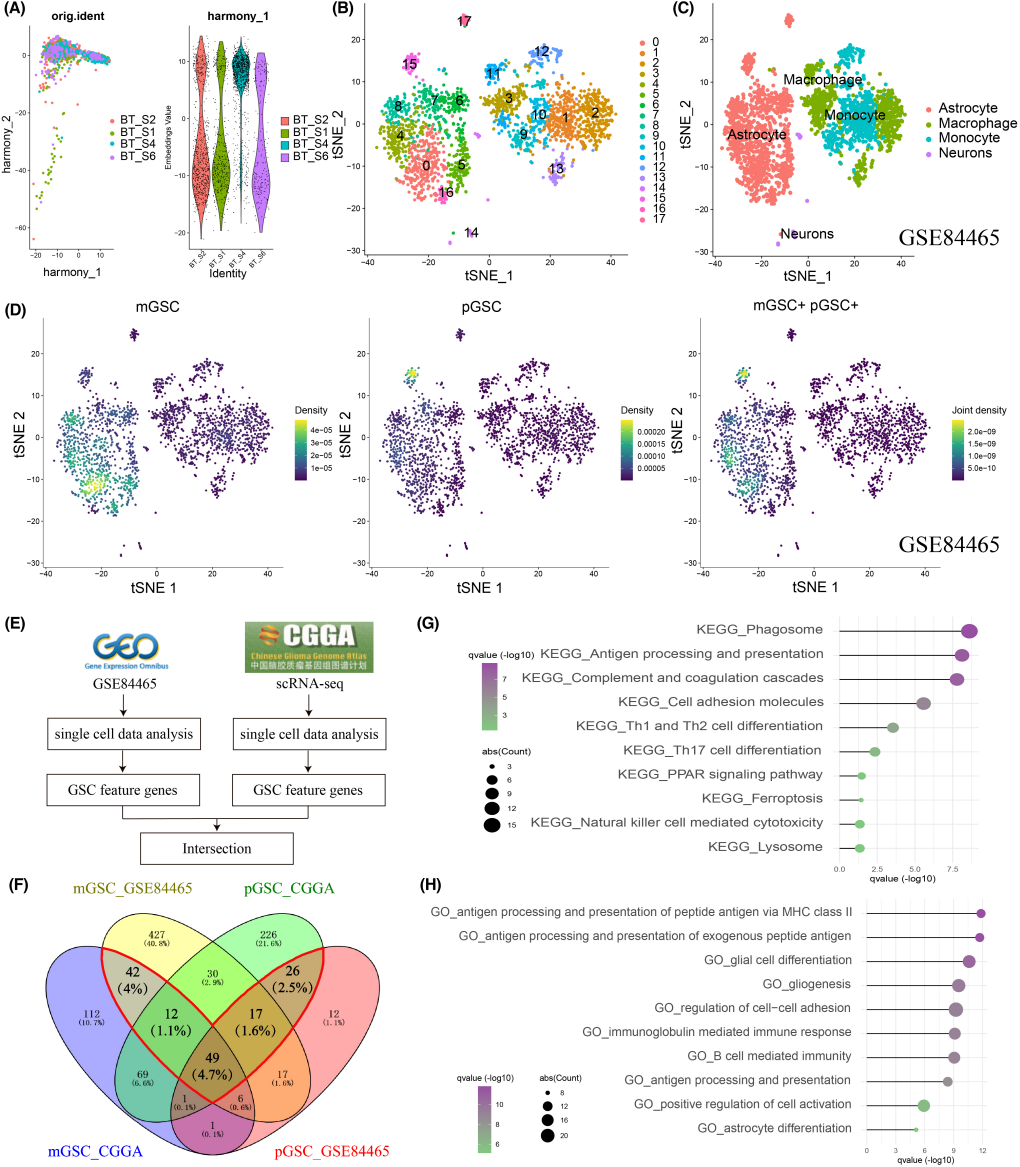
Identification of Glioma Stem Cell (GSC)‐related genes by single‐cell RNA sequencing (scRNA‐seq) analysis. (A) Integration of multiple sample data from the training cohort using the R package harmony. (B) t‐SNE plots colored by various cell types in the training cohort. (C) Cells were annotated into four clusters using the R package SingleR in the training cohort. (D) GSCs were defined using the R package irGSEA in the training cohort. mGSC, mesenchymal GSC; pGSC, proneural GSC. (E) The flow chart shows how to gain GSC‐related genes. (F) Venn diagram of GSC‐related genes in the training and validating cohorts. Genes in the red polygon were the selected GSC related‐genes. (G) Kyoto Encyclopaedia of Genes and Genomes (KEGG) function enrichment for GSC‐related genes. (H) Gene ontology (GO) function enrichment for GSC‐related genes.

The design flow chart of this study was presented in Figure S1. We aimed to explore the prognostic and treatment value of GSCs in glioma by studying the GSC‐related genes using the scRNA‐seq. After data screening and integration, we gained gene expression profiles of 2343 cells from the tumor tissue of four glioma training samples (BT_S1, BT_S2, BT_S4, and BT_S6) and 2916 cells from the tumor tissue of eight glioma validating samples (GS1, GS2, GS3, GS5, GS6, GS11, and GS12, GS13) (Figure 1A and Figure S2). 18 cell clusters in the training cohorts and 19 cell clusters in the validating cohorts were identified (Figure 1B and Figure S2). Six types of cells, including astrocyte, macrophage, monocyte, neuron, neutrophil, and T cell, were annotated and visualized in both training and validating cohorts (Figure 1C and Figure S2). Then, we explored the GSC distributions and found GSCs were mainly located at the astrocyte regions (mGSC: 0 cluster in the training cohort and 0, 3, 4, 6, 8, 9, and 12 clusters in the validating cohort; pGSC: 15 clusters in the training cohort and one cluster in the validating cohort; Figure 1D and Figure S2). Subsequently, we took the gene intersection between the training and validating cohorts and gained 146 GSC marker genes in gliomas (Figure 1E,F). In the KEGG pathway analysis, the most enriched were phagosome, antigen processing and presentation, th1 and th2 cell differentiation, PPAR signaling pathway, and so on (Figure 1G). GO enrichment analysis showed that these marker genes were notably enriched during antigen processing and presentation, immunoglobulin‐mediated immune responses, glial cell differentiation, gliogenesis, astrocyte differentiation, and others (Figure 1H).

### 3.2 GSC‐based grouping via machine learning

**FIGURE 2:**
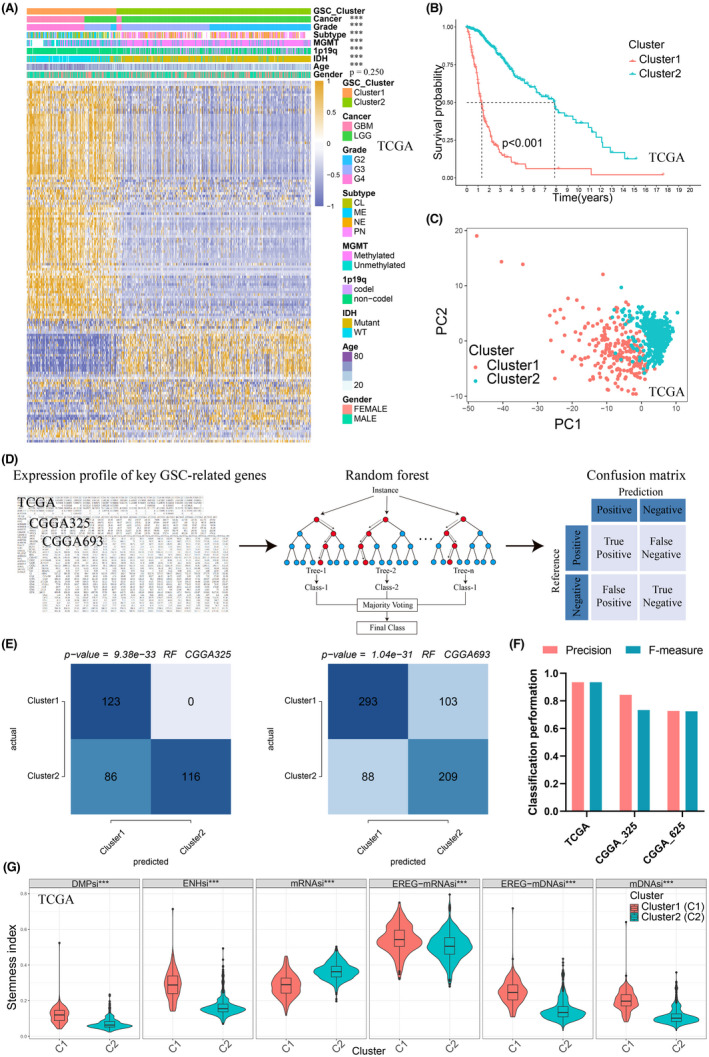
Machine learning for clustering and validation based on GSC‐related genes. (A) Heatmap demonstrated good separation of the two clusters calculated by non‐negative matrix factorization (NMF). (B) Kaplan–Meier survival analysis of the two clusters. Cluster 1 had a worse prognosis than cluster 2. (C) Principal component analysis (PCA) plot showed the two clusters could be discriminated clearly. (D) Schematic diagram of the random forest (RF). We trained an RF classifier in the TCGA training cohort to predict GSC glioma subtypes in two CGGA validating cohorts. (E) Validation of clustering by the RF algorithm in CGGA325 and CGGA693. Contingency tables displayed the clustering consistency between training and validating cohorts. (F) Classification performance of GSC clusters in the training and validating cohorts. *F*‐score, weight average of *F*‐scores. (G) Comparison of stemness index in the two clusters. Cluster 1 had a higher expression level of stemness index than cluster2. C1, cluster1; C2, cluster2.

Based on the expression profiles of 146 GSC marker genes, NMF was used to divide glioma patients in the TCGA cohort. The optimal cluster number was 2 (Figure S2). Heatmap showed that the two groups had distinct patterns of clinical traits, like cancer type, grade, subtype, MGMT, 1p19q, IDH, and age (Figure 2A). Survival curves showed the worse survival rates for cluster 1 (Figure 2B). PCA analysis visualized the disparity between the two clusters (Figure 2C). Subsequently, we used 10‐fold cross‐validation (CV) to evaluate the grouping performance in the TCGA cohort and then predict the glioma subtypes in the two CGGA validating cohorts with the TCGA cohort as the training set (Figure 2D). The contingency table showed the grouping consistency between the validating and training cohorts (Figure 2E). For the validating cohorts, patients were also divided into the two clusters, and cluster 1 had a lower survival probability (FigureS2–H). The grouping precisions were 0.935, 0.844, and 0.727 in TCGA, CGGA325, and CGGA693, separately. *F*‐measures of the grouping were 0.935, 0.734, and 0.725 in TCGA, CGGA325, and CGGA693, separately (Figure 2F). In addition, we further investigated the stemness index. Cluster 1 had significantly higher levels of DMPsi, ENHsi, EREG‐mRNAsi, EREG‐mDNAsi, and mDNAsi and lower levels of mRNAsi (Figure 2G). This result indicated that cluster1 was the GSCs‐enriched cluster.

### 3.3 Generation of risk scores and construction of prognostic models

**FIGURE 3:**
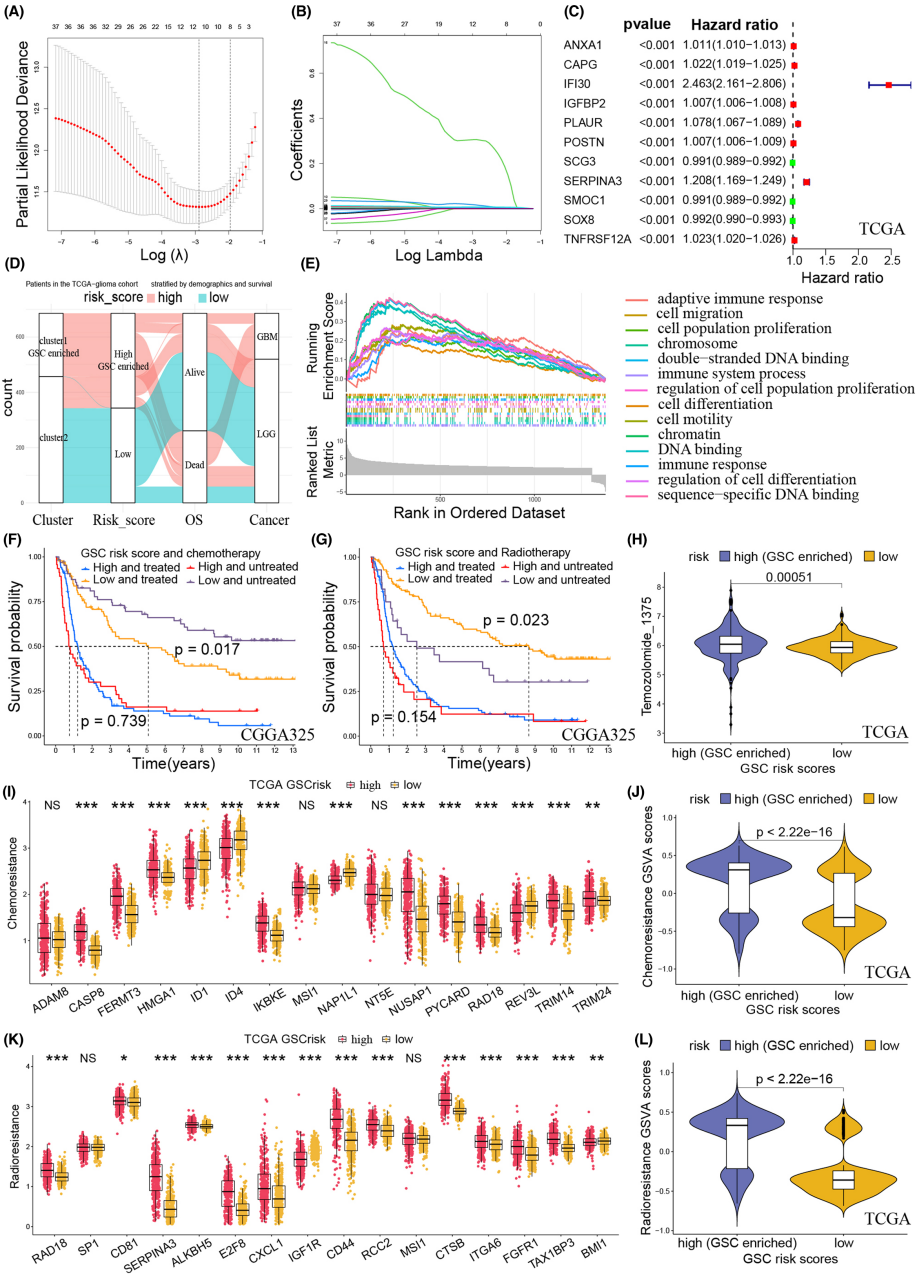
Construction of GSC risk scores and prediction of chemo‐radiotherapy resistance. (A) Coefficient profiles of the least absolute shrinkage and selection operator (LASSO) regression model. (B) Cross‐validation of tuning parameter screening in the LASSO regression model. (C) Univariate logistic regression identified 11 GSC genes' hazard ratios (HRs) and 95% confidence intervals (CIs) after LASSO regression filtration in the TCGA training cohort. (D) Sankey diagram showing high consistency between GSC‐associated clusters and risk scores. (E) Gene Set Enrichment Analysis (GSEA) function enrichment for the differentially expressed genes between the two risk‐score groups. (F) Kaplan–Meier curves of risk scores in patients receiving and not receiving chemotherapy in the CGGA325 cohort. (G) Kaplan–Meier curves of risk scores in patients receiving and not receiving radiotherapy in the CGGA325 cohort. For the low‐risk scores, there were significantly different survival rates between the patients receiving and not receiving chemo‐radiotherapy. For the high‐risk scores, the disparity of survival rates disappeared. (H) IC_50_ values of temozolomide in the two risk scores for the TCGA cohort. Patients in high‐risk scores had higher IC_50_ values of temozolomide. (I) Expression of chemoresistance feature genes in the two risk scores for the TCGA cohort. (J) Gene Set Variation Analysis (GSVA) scores of chemoresistance in the two risk scores for the TCGA cohort. Patients in high‐risk scores had higher chemoresistance. (K) Expression of radioresistance feature genes in the two risk scores for the TCGA cohort. (L) GSCA scores of radioresistance in the two risk scores for the TCGA cohort. Patients in high‐risk scores had higher radioresistance.

Considering that the number of 146 marker genes was too large in the GSCs grouping, GSCs risk scores were constructed using by fewer marker genes. First, we gained 38 differentially expressed GSC genes between the two clusters. Next, we imported the 38 genes into the LASSO regression, gained 11 GSCs‐related prognostic genes, and built a risk score model (Figure 3A,B, and Figure S3). Univariate Cox regression showed that ANXA1, CAPG, IFI30, IGFBP2, PLAUR, POSTN, SERPINA3, and TNFRSF12A were detrimental to prognosis, whereas SCG3, SMOC1, and SOX8 were beneficial for patients' survival (Figure 3C, and Figure S3). Sankey plot demonstrated high consistency between GSCs‐associated clusters and risk scores (Figure 3D). This result indicated high GSCs‐risk‐score gliomas were the GSCs‐enriched gliomas. Gene Set Enrichment Analysis (GSEA) analysis showed that GO terms like cell migration, cell population proliferation, cell differentiation, DNA binding, and immune response were more active in patients with higher risk scores (Figure 3E).

Then, the GSC risk scores were used to build a prognostic model. Survival curves revealed a good prognosis separation of patients with high‐ and low‐risk scores (Figure S3). A nomogram prognosis model was constructed using independent prognostic factors, including GSC risk score, pathological grade, and patient age (Figure S4). Calibration plots indicated that observed and predicted probabilities for 1‐, 3‐, and 5‐year overall survival (OS) had excellent concordance (Figure S4). ROC curves further confirmed the predictive ability of the nomogram (AUC, area under the curve: 0.907 in TCGA, 0.871 in CGGA325, and 0.742 in CGGA693) and included variables in both the training and validating cohorts (Figure S4).

### 3.4 Chemo‐radiotherapy resistance of GSC‐stratified groups

**FIGURE 4:**
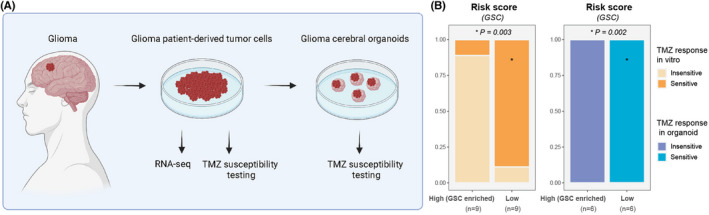
Temozolomide sensitivity validation for risk scores. (A) Schematic of major stages in the TMZ susceptibility experiments. (B) Bar chart showing the proportions of TMZ‐sensitive responses between the patients with high‐ and low‐risk scores. Patients with high‐GSC‐risk scores had significantly lower proportions of TMZ‐sensitive response.

The survival curves of the low‐risk glioma patients showed significant discrimination of survival probabilities in the patients with or without chemo‐radiotherapy. However, the high‐risk patients did not display the statistical disparity of survival probabilities in this type of comparison (Figure 3F,G). Next, we predicted the therapy response of TMZ and found that patients with high‐risk scores had higher resistance (Figure 3H and Figure S5). The comparison of feature‐gene expressions also revealed that high‐GSCs‐risk patients had significantly higher risk of chemoresistance and radioresistance than those with low‐risk scores (Figures 3I–L and Figure S5). In addition, both TMZ sensitivity experiments in vitro and organoids indicated that patients with high‐risk scores had significantly lower incidence of TMZ sensitive response (Figure 4B).

### 3.5 Prediction of TTF sensitivity for GSCs‐stratified groups

**FIGURE 5:**
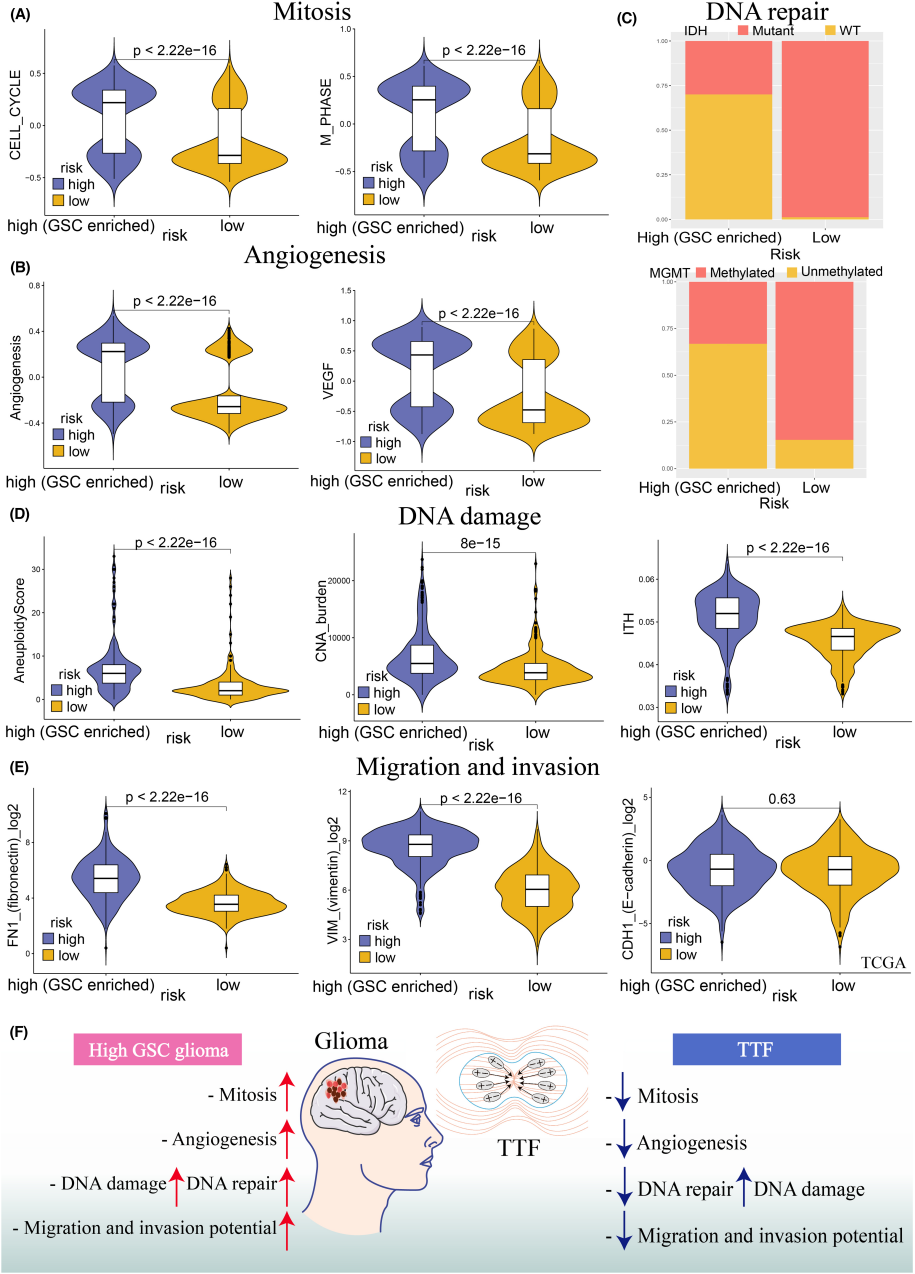
Risk scores predict tumor‐treating field (TTF) sensitivity in the TCGA training cohort. (A) GSVA scores of the mitosis (cell cycle and M phase) between the two risk scores. (B) GSVA scores of the angiogenesis (angiogenesis and VEGF molecules) between the two risk scores. (C) Bar chart showing the proportions of DNA repair (IDH wildtype and MGMT unmethylation) between the two risk scores. (D) DNA damage level, including aneuploidy score, somatic copy number alternations (CNA) burden, and intratumor heterogeneity (ITH) between the two risk scores. (E) Migration and invasion potential (fibronectin, vimentin, and E‐cadherin) between the two risk scores. (F) Schematic diagram showing the relationship between the high‐GSC‐risk‐score gliomas and TTFs. Gliomas in the high‐risk‐score group had a higher level of mitosis, angiogenesis, DNA damage, DNA repair, migration, and invasion potential. TTF could inhibit the occurrence and development of high‐GSC‐risk‐score gliomas by impairing mitosis, angiogenesis, DNA repair, migration and invasion potential, and increasing DNA damage.

We investigated the sensitivity of gliomas to TTF therapy in terms of mitosis, angiogenesis, DNA repair, DNA damage, migration, and invasion. For mitosis, the high‐risk group had higher expressions of cell cycle and M phase than the low‐risk group (Figure 5A, Figure S6). In terms of angiogenesis, we found more activated VEGF and angiogenesis pathways in the high‐risk group (Figure 5B and Figure S6B). Wild‐type IDH and unmethylated MGMT, indicating a higher DNA repair capacity, were more frequently observed in the high‐risk group (Figure 5C and Figure S6C). DNA damages, including aneuploidy, CNA burden, and ITH, were all more pronounced in the high‐risk score group (Figure 5D). In addition, we found that the high‐risk group exhibited a significantly higher expression level of FN1 and VIM, which indicated glioma in this group was easier to migrate and invade (Figure 5E and Figure S6D). In summary, TTF may target GSCs‐enriched gliomas by inhibiting glioma mitosis, angiogenesis, DNA repair, migration, invasion, and increasing DNA damage (Figure 5F).

### 3.6 Efficacy of TTFs in preventing GBM recurrence

**FIGURE 6:**
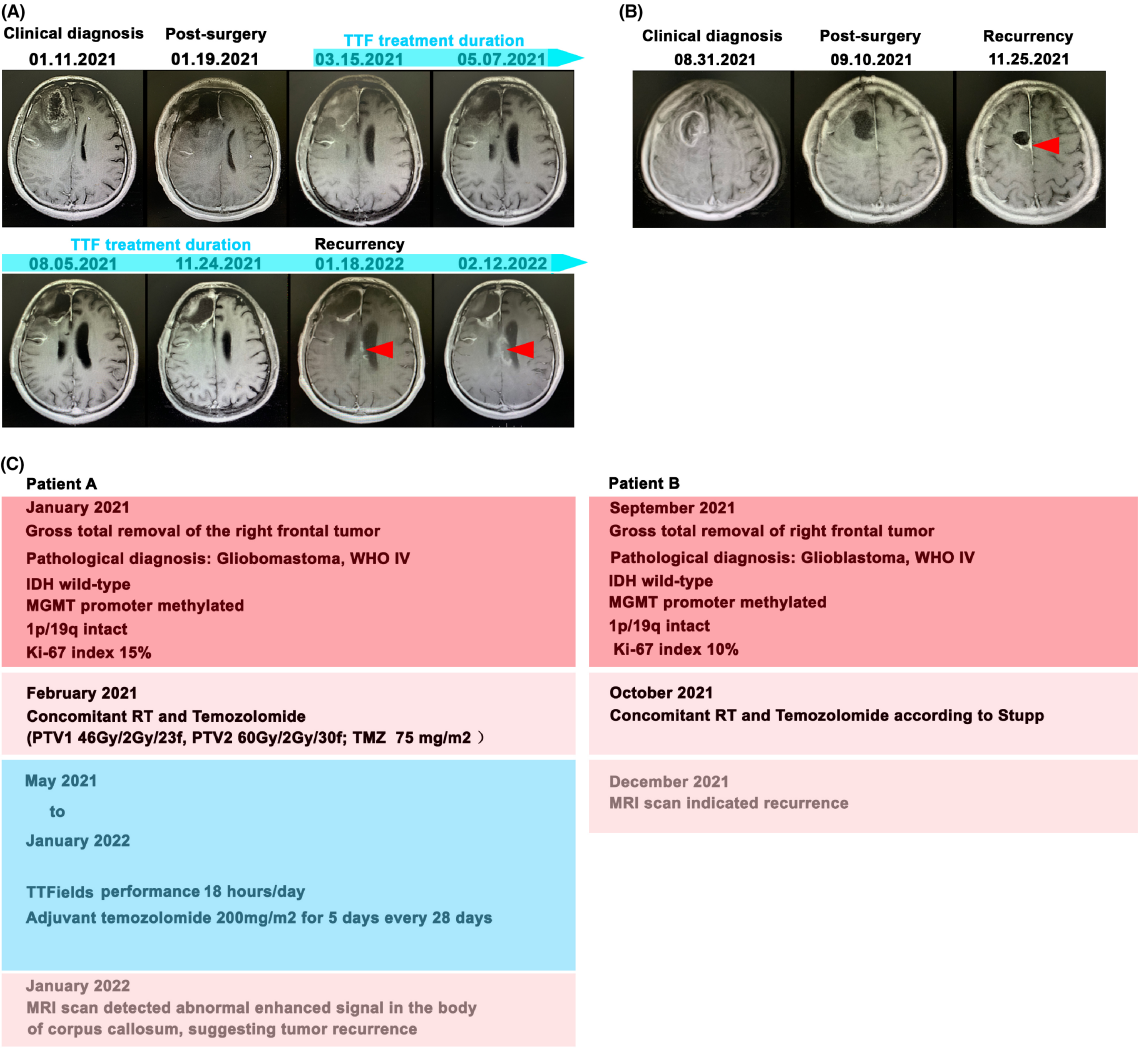
Case study of glioblastoma (GBM) patients who may benefit from TTF. (A) Patient A was initially diagnosed with GBM. “01.11.2021 MRI” indicated right frontal lesion with enhanced signal. Lesion was surgically removed under right frontal craniotomy: “01.19.2021 MRI”. The follow‐up MRI indicated that TT fields therapy was administered 3 months after surgery: “03.15.2021–11.24.2021 MRI”. No recurrence was detected by routine surveillance scans until 9 months after surgery. (B) Patient B was analogously detected the right frontal lesion and diagnosed with GBM. Lesion was surgically removed under right frontal craniotomy: “09.10.2021 MRI”. Unfortunately, recurrency was detected in “11.25.2021 MRI”. (C) Clinical features of patient A and B were demonstrated. Red label: initial diagnosis; Pink label: post‐surgical treatment; Blue label: no recurrency periods; Light pink label: recurrency.

We further investigated the efficacy of TTFs in patients with GBM. A phase I, open‐label study of TTFs was conducted to estimate whether 150 kHz is safe for GBM patients. The TTFs were equipped more than 18 h each day. To date, patient A presented with progressive left limb weakness and memory loss for 3 months. MRI indicated a right frontal lesion with an enhanced signal. Consequently, the patient underwent a right frontal craniotomy. Pathological examination confirmed GBM (WHO IV) with wild‐type IDH and a methylated MGMT promoter. The patient received standard radiotherapy and concomitant chemotherapy. TTF therapy was administered 4 months after surgery. No recurrence was detected on routine surveillance scans until 12 months after surgery (Figure 6A,C). Patient B experienced with headache for 2 months. MRI scan revealed a the right frontal lesion. Craniotomy was recommended, and Patient B underwent gross total resection of the lesion. Pathological examination confirmed the diagnosis of GBM (WHO IV) with wild‐type IDH and a methylated MGMT promoter. The patient received standard radiotherapy and concomitant chemotherapy (Figure 6B,C). Multimodel MRI was performed 3 months after surgery and showed tumor recurrence. This clinical performance indicated that TTFs may improve the disease profiles of GBM patients and may contribute to prolong the progression‐free survival (PFS).

### 3.7 Tumor microenvironment and clinical traits of the GSCs‐stratified groups

In addition, we investigated the tumor microenvironment of the two clusters using Xcell, CIBERSORT, and estimate algorithms. Xcell algorithms showed the disparity of 64 immune and stromal cell types between the two clusters (Figures S7 and S8). CIBERSORT algorithms indicated that cluster 1 had higher expression levels of T cells CD8, T cells follicular helper, T cells regulatory (Tregs), macrophages M0, and neutrophils, and lower expression levels of B cells memory, NK cells activated, and monocytes than the cluster2 (Figures [Link], [Link], and S10). Estimate algorithms showed increased stromal and immune scores and decreased tumor purity in cluster 1 (Figures [Link], [Link], and S10). Subsequently, the immune checkpoints were compared between the two clusters. Antigens, cell adhesions, co‐stimulators, ligands, receptors, co‐inhibitors, and other immune checkpoints were all overexpressed in cluster 1 (Figures [Link], [Link], and S10).

The clinical traits of the two clusters were also different. The cluter1 had significantly higher pathological grades than the cluster 2 (Figure S11). Samples with unmethylated MGMT, 1p19q non‐codeletion, and wildtype IDH significantly accounted for the majority of the cluster 1 (Figure S11). The proportions of samples with more malignant Classical (CL) and Mesenchymal (ME) in cluster1 were higher than those in cluster2 (Figure S11).

### 3.8 Genomic features of GSC‐stratified groups

Somatic mutations and CNAs were investigated between the two clusters, based on the TCGA dataset. Cluster1 had significantly higher arm‐and focal‐level amplification frequencies in chromosomes 7p and 7q and higher deletion frequencies in chromosomes 10p and 10q (Figures S12). Mutations were more common in cluster1 than cluster2, like 3′ UTR, 3′ flank, 5′ flank, 5′ UTR, in‐frame insertion, introns, missense, nonsense, nonstop, RNA, silent, splice region, and splice site mutations. The frequencies of single‐nucleotide variations (SNVs), insertions, and deletions presented higher in cluster 1 than cluster 2. Among the detected SNVs, the C > T and G > A were the most common mutations in cluster1 (Figure S12).

The Waterfall plot showed mutation landscapes with significantly different frequencies in the two clusters (Figure S12). Cluster 1 had higher frequent mutations in PTEN, EGFR, SPTA1, TTN, RB1, NF1, FLG2, GRM3, DNAH3, and other genes. Cluster2 had higher frequent mutations in IDH1, CIC, TP53, ATRX, NOTCH1, FUBP1, ARID1A, IDH2, NIPBL, and other genes (Figure S12). In the two clusters, the strongest co‐occurrent mutation pairs were TP53‐ATRX, and the strongest mutually exclusive mutation pairs were IDH1‐EGFR (Figure S12). GO biological process analysis found that these highly frequent mutated genes were mainly enriched in glial cell proliferation, neuroblast proliferation, and so on (Figure S12).

### 3.9 Prediction of immunotherapy response of GSC stratified groups

Considering the apparent disparities in the immune microenvironment between the two clusters, immunotherapy that targets GSCs was investigated. The high‐risk group exhibited higher expression levels of immune checkpoints, including antigen presence, cell adhesion, co‐stimulator, ligand, receptor, co‐inhibitor, and others (Figures S13 and S14A,B). Moreover, the high‐risk group also had a decreased level of microsatellite instability (MSI) and an increased level of SNV neoantigens (Figure S13). In addition, immune subtype analysis showed a significantly higher proportion of lymphocyte‐depleted subtype (immune cold microenvironment) in patients with high‐risk scores (Figure S13).

## 4 DISCUSSION

As the “ethnic minority” of glioma‐initiating cells, GSCs exerts its crucial role in chemo‐radiotherapy resistance. During chemo‐radiotherapy procedure, pGSCs can be transformed into mGSCs that demonstrates stronger resistance.^8^ In recent years, new therapy approaches targeting GSCs have been widely investigated, such as metabolic therapy, immunotherapy, and anti‐angiogenesis therapy.^8^ However, compared with other cancers, clinical trials investigating gliomas did not show optimistic outcomes.^65^, ^66^, ^67^ On the contrary, glioma patients were not screened for individual factors, like immune checkpoints, tumor microenvironment, mismatch repair deficiency, and others, which may influence the therapeutic efficiency.^68^, ^69^ However, some therapeutic drugs may indeed work poorly owing to the existence of the blood–brain barrier and the like. Therefore, it is an urgent need to systematize the glioma GSCs subtypes and explore new therapeutic approaches for GSC resistance, such as TTFs.

Currently, in an in silico study, we novelty initiated and extracted the GSC‐related marker genes in gliomas using scRNA‐seq. Based on these marker genes, machine learnings were adopted for patient grouping and validation. An extended annotation of the stemness index, function enrichment, immune microenvironment, and genomic alterations was carried out for GSC‐related patient groups. Risk scores, based on the differentially expressed GSC genes between GSC‐stratified groups, were generated by Lasso and Cox regressions. Then, the risk scores were used to construct a nomogram prognostic model. Furthermore, chemo‐radiotherapy resistance, immunotherapeutic response, and TTF sensitivity were predicted based on the GSC risk scores.

The two GSCs clusters had distinct biological features. Patients in cluster 1 with worse prognosis had higher proportions of MGMT promoter unmethylation, 1p19q noncodeletion, IDH wildtype, CL and ME subtypes, all of which correlated with the malignant phenotype. Besides, cluster1 had higher stemness indices, which indicated increased GSCs enrichment. Notably, despite the relatively high immune scores, patients in cluster 1 had higher expression of the immune checkpoint molecule programmed death‐ligand 1 (PD‐L1) and immunosuppressive cells, such as regulatory T cells (Tregs) and M2 macrophages. Previous studies have found that GSCs could recruit anti‐inflammatory M2 macrophages and induce Treg expansion to suppress both innate and adaptive immune responses.^70^ Additionally, cell‐to‐cell contact between GSCs and immunocytes mediated by PD‐L1 can inhibit the immune cell function.^70^ Overall, compared with cluster 2, gliomas in GSCs‐enriched cluster1 exhibited a immunosuppressive microenvironment.

Based on the profiles of differentially expressed GSCs genes between the two clusters, we calculated GSCs‐related risk scores. The risk scores exhibited high efficiency to predict glioma patients' survival outcomes. Among the 11 genes with risk scores, ANXA1, encoding a membrane‐localized protein, plays an important role in stem cell maintenance and growth.^71^ Johnstone et al. indicated that ANXA1 was required for cancer initiation and cancer stem cell (CSC) maintenance in breast cancer.^72^ Geary et al. demonstrated that fibroblast‐secreted ANXA1 induced prostate tumor cells to gain stem‐cell‐like traits.^73^ Insulin‐Like Growth Factor Binding Protein 2, encoded by IGFBP2 gene, is highly expressed in GSCs‐high‐risk gliomas. One study showed that IGFBP2 promoted self‐renewal and proliferation of neural stem cells and inhibited their differentiation to neurons and astrocytes.^74^ In another study, IGFBP2 was identified to induce GBM pathogenesis through GSCs enrichment.^75^ Periostin (POSTN) is a secreted extracellular matrix protein that functions in stem‐cell maintenance and metastasis. Qin et al. found that POSTN promoted adipose‐derived stem cell adhesion and migration in the disease of hind limb ischemia.^76^ Zhou et al. suggested that POSTN secreted by GSCs can recruit M2 macrophages and further promote glioma growth.^77^ Overall, the aforementioned studies supported the idea that these risk‐score genes were crucial to maintain GSCs growth and induce the occurrence of GSCs‐enriched gliomas.

GSCs’ resistance to chemoradiotherapy was increasingly recognized. GSCs overexpressed ATP‐binding cassette transporters (ABC) and MGMT to achieve multidrug resistance.^78^, ^79^ Additionally, GSCs radioresistance was conferred by hypoxia‐mediated activation of the DNA damage checkpoint response.^80^ In our study, patients with GSCs high‐risk scores exhibited higher chemo‐radiotherapy resistance and lost the survival disparity in determining whether to receive chemo‐radiotherapy or not. Therefore, we investigated other therapy approaches, like TTFs, in patients with different GSCs risk scores.

TTFs is an emerging treatment in the field of tumor treatment, with a low side‐effect and resistance. Different from the systematic toxicity of chemotherapeutics, the side‐effect of TTFs mainly focused on local adverse skin effects, like dermatitis, erosin, and others.^12^ A phase 3 randomized clinical study showed that only 14% of patients treated with TTFs had tumor resistance, which could also be reversed by reducing TTFs frequency.^12^, ^81^ In addition, the National Comprehensive Cancer Network (NCCN) has recommended TTFs to treat recurrent or drug‐resistant tumors.

TTFs can effectively inhibit glioma growth and improve the survival outcomes, especially for the GBM.^64^, ^82^, ^83^ Some potential TTFs therapy targets, including tumor mitosis, angiogenesis, DNA repair, DNA damage, migration, and invasion. TTFs can inhibit mitosis in fast‐proliferating tumor cells, thus leading to abnormal chromosome separation, multinucleation, and apoptosis.^13^ DNA is another therapeutic target of TTFs. TTFs can induce tumor cell death by inhibiting the DNA repair and aggravating DNA damage.^13^ In addition, growing evidence suggests that TTFs inhibits tumor angiogenesis by downregulating VEGF and HIF1α expression.^84^ TTFs also impairs the invasion and migration capacities of tumor cells by downregulating vimentin, E‐cadherin, and fibronectin expression.^84^, ^85^ In this study, we investigated the role of TTFs in patients with different GSCs risk scores. We found that high GSCs‐risk‐score gliomas had enhanced levels of mitosis, angiogenesis, DNA damage, migration, and invasion potential. Notably, all of these pathological processes can be inhibited by TTFs. Additionally, previous studies have initially investigated the relationship between GSCs and TTFs. TTFs could induce GSCs autophagy and act synergistically with immunotherapy targeting GSCs to reduce chemoresistance.^15^, ^16^

Therefore, we boldly speculated that TTF is a promising therapy targeting GSC‐enriched gliomas. TTFs can prevent the occurrence and progression of GSCs‐enriched gliomas by inhibiting mitosis, angiogenesis, migration, and invasion. Besides, TTFs disrupted the original DNA balance in GSC‐enriched gliomas by decreasing DNA repair and increasing DNA damage (Figure 5F).

Considering the apparent disparities of the immune microenvironment between the two GSCs clusters, we also explore the immunotherapy response. Although, some immunotherapies have been identified to target GSCs,^86^ the effects of immunotherapy were unclear in our study. Patients with high GSCs risk scores had higher expression levels of immune checkpoint molecule PD‐L1, which induced the immunosuppressive context.^87^ Lymphocyte Depleted immune subtypes (immune cold microenvironment) and neoantigens (antigen presentation capacity) were also observed in the high‐risk score group, which represented a better immunotherapy response.^88^ By contrast, high‐risk‐score patients had lower MSI levels, indicating a worse response to immunotherapies.^89^ This finding may require further investigation using the glioma immunotherapy cohort.

Although our study initiated some innovative perspectives, its limitations urgently require further evaluations. One major limitation was the lack of external real‐world data to confirm and support our findings. Another limitation was the lack of large glioma cohorts to predict the TTFs therapy response. In addition, the detailed mechanism of GSCs in TTF responses remained to be elucidated.

## 5 CONCLUSION

Our study constructed a GSCs signature consisting of 11 GSCs‐specific genes and identified its prognostic value in gliomas. Our results proved that TTFs was a promising therapeutic approach for GSCs‐enriched glioma patients.

## AUTHOR CONTRIBUTIONS

B.Chen, LY Zhang and CT Li designed and drafted the manuscript, TTF clinical organization; B. Chen, LY. Zhang and CT Li organized figures and edited legends. B Chen, XX Zhou, LT Yang, HS Zhou, M Meng and CT Li revised the article; B. Chen, LY. Zhang and CT Li conducted data analysis; XX Zhou, CT Li, H Wu, and ZX Liu organized patients with the treatment of TTF.

## FUNDING INFORMATION

This work was supported by the Nature Science Foundation of China (no. 81402249 to L.Y. Zhang), the Natural Science Foundation of Hunan Province (no. 2019JJ50963 to L.Y. Zhang), and Fundamental Research Funds for the Central Universities of Central South University (no. 160171016 to B. Chen).

## CONFLICT OF INTEREST

All authors declare no conflict of interest.

## Supporting information


Figure S1

Figure S2

Figure S3

Figure S4

Figure S5

Figure S6

Figure S7

Figure S8

Figure S9

Figure S10

Figure S11

Figure S12

Figure S13

Figure S14

## Data Availability

The data for bioinformatic analysis that support the findings of this study are available in open‐source TCGA, CGGA, and GEO datasets. The TTF clinical data are available upon reasonable request.
