# Supplementary material for: Glioma stem cell signature predicts the prognosis and the response to tumor treating fields treatment
Source: CNS Neurosci Ther. 2022 Sep 7;28(12):2148–62. doi: 10.1111/cns.13956 (PMC9627385; doi:10.1111/cns.13956)
Supplement: Supplementary file 1 — Figure S1 [file CNS-28-2148-s004.pdf]

Supporting information

Figure S1. The flow chart of data analysis.

Figure S2. scRNA-seq analysis for identifying GSC-related genes and machine learning for validating clustering in the CGGA validating cohort. (A) Integration of multiple sample data using the R package harmony. (B) t-SNE plots colored by various cell types. (C) Cells were annotated into 4 clusters using the R package SingleR. (D) GSCs were defined using the R package irGSEA. (E) Cluster stability analysis by NMF algorithms. (F) Heatmaps showed that samples from cluster1 and cluster2 had different demographical features, tumor pathologies, and mutation status. (G) Kaplan-Meier survival analysis of the two clusters. Cluster1 had a worse prognosis than cluster2. (H) PCA plots showed that the two clusters could be discriminated clearly.

Figure S3. Construction of risk scores and prognosis analysis. (A) Univariate logistic regression showed 11 GSC genes' hazard ratios (HRs) and 95% confidence intervals (Cls) after LASSO regression filtration in the CGGA325 and CGGA693 validating cohorts. (B) Calculating risk scores and dividing high- and low-risks. (C) Survival curves of risk scores in pan-glioma, low-grade glioma (LGG), and GBM groups for the CGGA325 validating cohort. (D) Survival curves of risk scores in pan-glioma, LGG, and GBM groups for the CGGA693 validating cohort. (E) Survival curves of risk scores in pan-glioma and LGG groups for the TCGA training cohort. Patients in the high-risk-score group had a worse prognosis.

Figure S4. Construction and evaluation of the nomogram prognosis model. (A) A nomogram integrating GSC risk score, glioma grade, patient age, and overall survival probability. (B) Calibration plots showing the correlation between actual and predicted overall survival rates in the TCGA training cohort. (C) ROC curve plot evaluating the nomogram model, GSC risk score, age, and grade in the TCGA training cohort. (D) Calibration plots showing the correlation between actual and predicted overall survival rates in the CGGA325 and CGGA693 validating cohorts. (E) ROC curve plots evaluating the area under the curve (AUC) values of the nomogram prognosis model in the CGGA325 and CGGA693 validating cohorts. (F) ROC curve plots evaluating the

AUC values of GSC risk score, age, and grade in the nomogram model for the CGGA325 and CGGA693 validating cohorts.

Figure S5. Prediction of chemo-radiotherapy resistance in the CGGA325 and CGGA693 validating cohorts. (A) Expression of chemoresistance feature genes in the two risk scores. (B) GSVA scores of chemoresistance in the two risk scores. Patients in high-risk scores had higher chemoresistance. (C) Expression of radioresistance feature genes in the two risk scores. (D) GSVA scores of radioresistance in the two risk scores. Patients in high-risk scores had higher radioresistance. (E) IC50 values of temozolomide in the two risk scores. Patients in high-risk scores had higher IC50 values of temozolomide.

Figure S6. Prediction of TTF sensitivity in the CGGA325 and CGGA693 validating cohort. (A) GSVA scores of the mitosis (cell cycle and M phase) between the two risk scores. (B) GSVA scores of the angiogenesis (angiogenesis and VEGF molecules) between the two risk scores. (C) The bar chart showing the proportions of DNA repair (IDH wildtype and MGMT unmethylation) between the two risk scores. (D) The migration and invasion potential (fibronectin, vimentin, and E-cadherin) between the two risk scores. Gliomas in the high-risk-score group had a higher level of mitosis, angiogenesis, DNA repair, migration and invasion potential, which could be inhibited by TTFs.

Figure S7. Immune characteristics of the two GSC clusters. (A) The heatmap visualized the expression levels of 64-type cells calculated by the xCell algorithm and GSC clusters in the TCGA training cohort. (B) ESTIMATE-algorithm-calculated tumor purity, stromal scores, and immune scores of the two clusters in the TCGA training cohort. Cluster1 had a higher level of stromal scores, immune scores, and a lower level of tumor purity than cluster2. (C) Molecular levels of 7-type immune checkpoints in the two clusters for the TCGA training cohort. Cluster1 had a higher expression level of all immune checkpoints than cluster2.

Figure S8. Immune characteristics of the two GSC clusters. (A) Heatmaps visualized the expression levels of 64-type cells calculated by xCell algorithm and GSC clusters

in the CGGA325 and CGGA693 validating cohorts. (B) The expression level of 22 immunocyte types calculated by CIBERSORT algorithm in the two clusters for the TCGA training cohort.

Figure S9. Immune characteristics of the two GSC clusters. (A) The expression level of 22 immunocyte types calculated by CIBERSORT algorithm in the two clusters for the CGGA325 validating cohort. (B) ESTIMATE-algorithm calculated tumor purity, stromal scores, and immune scores of the two clusters in the CGGA325 validating cohort. Cluster1 had a higher level of stromal scores, immune scores, and a lower level of tumor purity than the cluster. (C) Molecular levels of 7-type immune checkpoints in the two clusters for the CGGA325 validating cohort. Cluster1 had a higher expression level of all immune checkpoints than cluster2.

Figure S10. Immune characteristics of the two GSC clusters. (A) The expression level of 22 immunocyte types calculated by CIBERSORT algorithm in the two clusters for the CGGA693 validating cohort. (B) ESTIMATE-algorithm calculated tumor purity, stromal scores, and immune scores of the two clusters in the CGGA693 validating cohort. Cluster1 had a higher level of stromal scores, immune scores, and a lower level of tumor purity than the cluster. (C) Molecular levels of 7-type immune checkpoints in the two clusters for the CGGA693 validating cohort. Cluster1 had a higher expression level of all immune checkpoints than cluster2.

Figure S11. Clinical features of the two GSC clusters in the TCGA training and CGGA validating cohorts. (A) The proportions of different tumor grades in the two clusters. (B) Samples with or without the MGMT promoter methylation in the two clusters. (C) Samples with or without the chromosome 1p/19q codeletion in the two clusters. (D) Samples with or without the IDH mutation in the two clusters. (E) The four GBM subtypes in the two clusters. Patients in cluster1 had a higher proportion of gliomas with enhanced grades, unmethylated MGMT, non-codel 1p19q, wildtype IDH, CL and ME subtypes.

Figure S12. Genomic alterations in the two GSC clusters in the TCGA training cohort. (A) Comparison of arm-level amplification and deletion frequencies between the two

clusters. (B) Comparison of focal-level amplification and deletion frequencies between the two clusters. (C) Comparison of variant types between the two clusters. (D) The waterfall plot showing the mutation landscapes with significantly different frequencies in the two clusters. (E) The forest plot listing the top 30 most mutated genes between the two clusters. (F) The heatmap showing the concurrence or mutual exclusivity of the top 30 most mutated genes. (G) GO function enrichment for the top 30 most mutated genes.

Figure S13. Prediction of immunotherapy response in the TCGA training cohort. (A) Molecular levels of 7-type immune checkpoints in the two risk scores. Patients in the high-risk-score group had a higher expression level of all immune checkpoints. (B) Microsatellite instability (MSI) in the two risk scores. Patients in the high-risk scores had lower MSI. (C) Neoantigens expression in the two risk scores. Patients in the high-risk scores had higher neoantigen expression. (D) Distribution of six immune subtypes in the two groups. Patients in the high-risk scores had a higher proportion of Lymphocyte Depleted subtypes.

Figure S14. Prediction of immunotherapy response in the CGGA325 and CGGA693 validating cohort. (A) Molecular levels of 7-type immune checkpoints in the two risk scores for the CGGA325 validating cohort. (B) Molecular levels of 7-type immune checkpoints in the two risk scores for the CGGA693 validating cohort. Patients in the high-risk-score group had a higher expression level of all immune checkpoints.
