# Supplementary figures and images for: Glioma stem cell signature predicts the prognosis and the response to tumor treating fields treatment
Source: CNS Neurosci Ther. 2022 Sep 7;28(12):2148–62. doi: 10.1111/cns.13956 (PMC9627385; doi:10.1111/cns.13956)

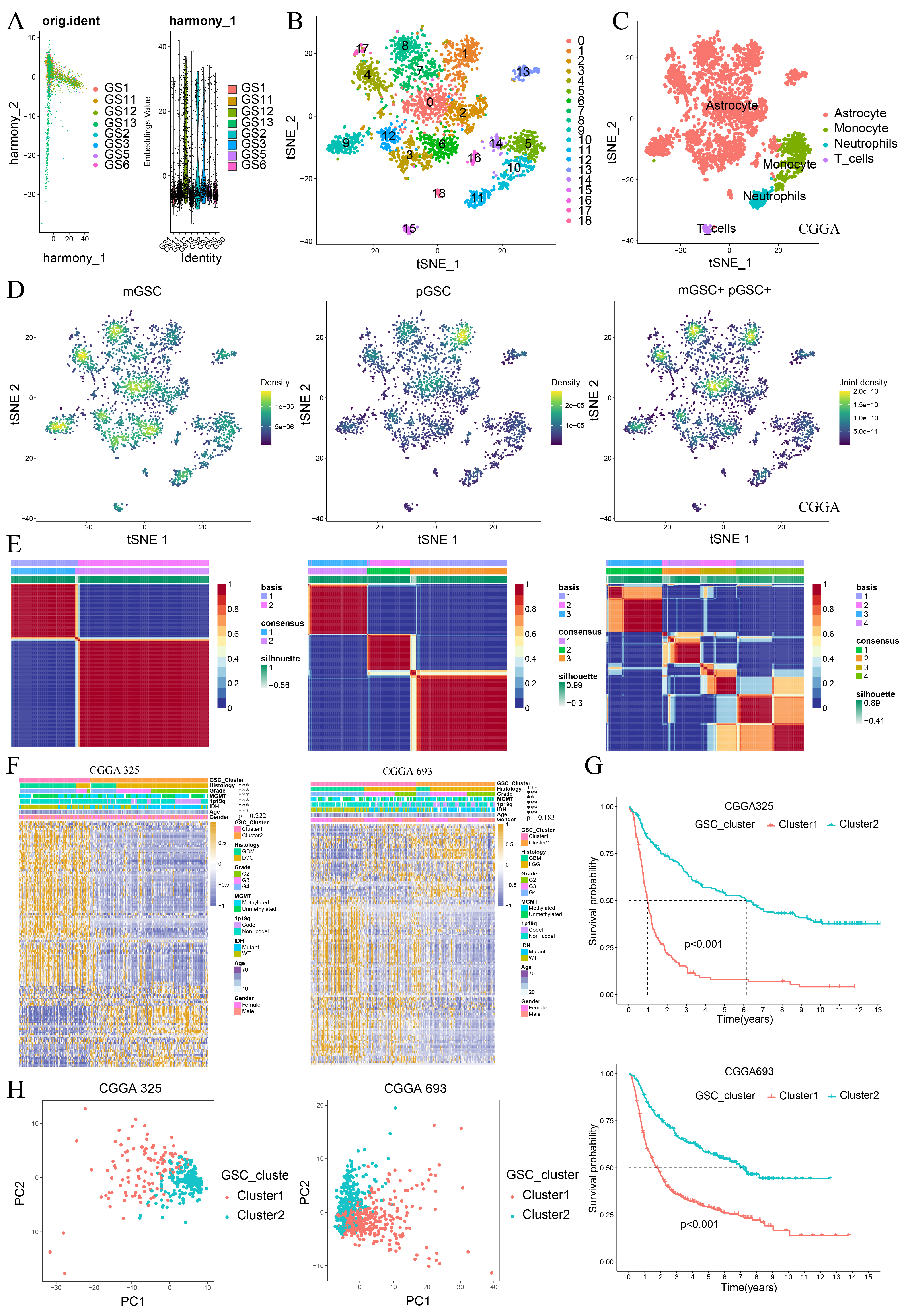

Supplement: Supplementary file 2 — Figure S2 [file CNS-28-2148-s013.jpg]

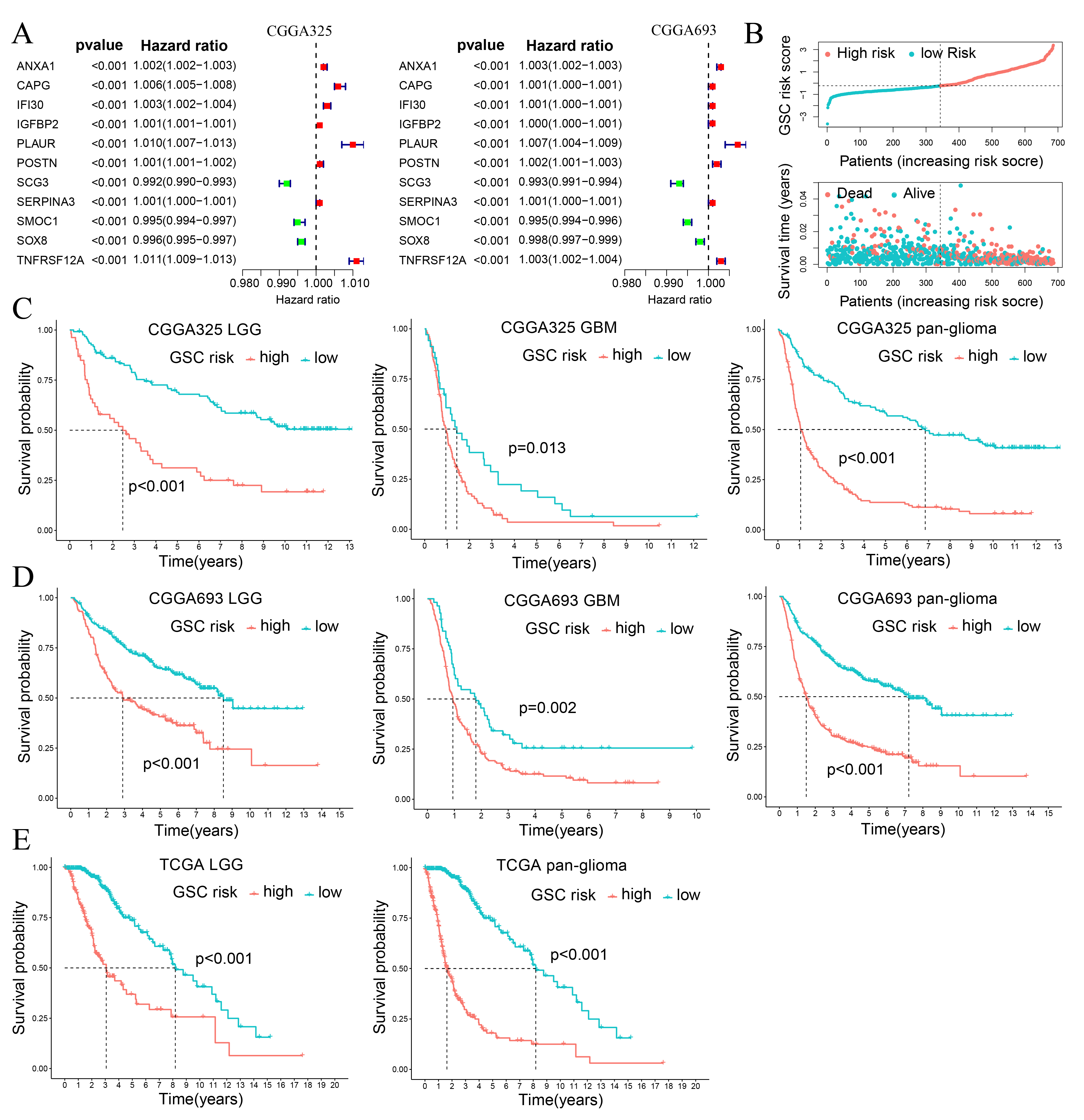

Supplement: Supplementary file 3 — Figure S3 [file CNS-28-2148-s008.jpg]

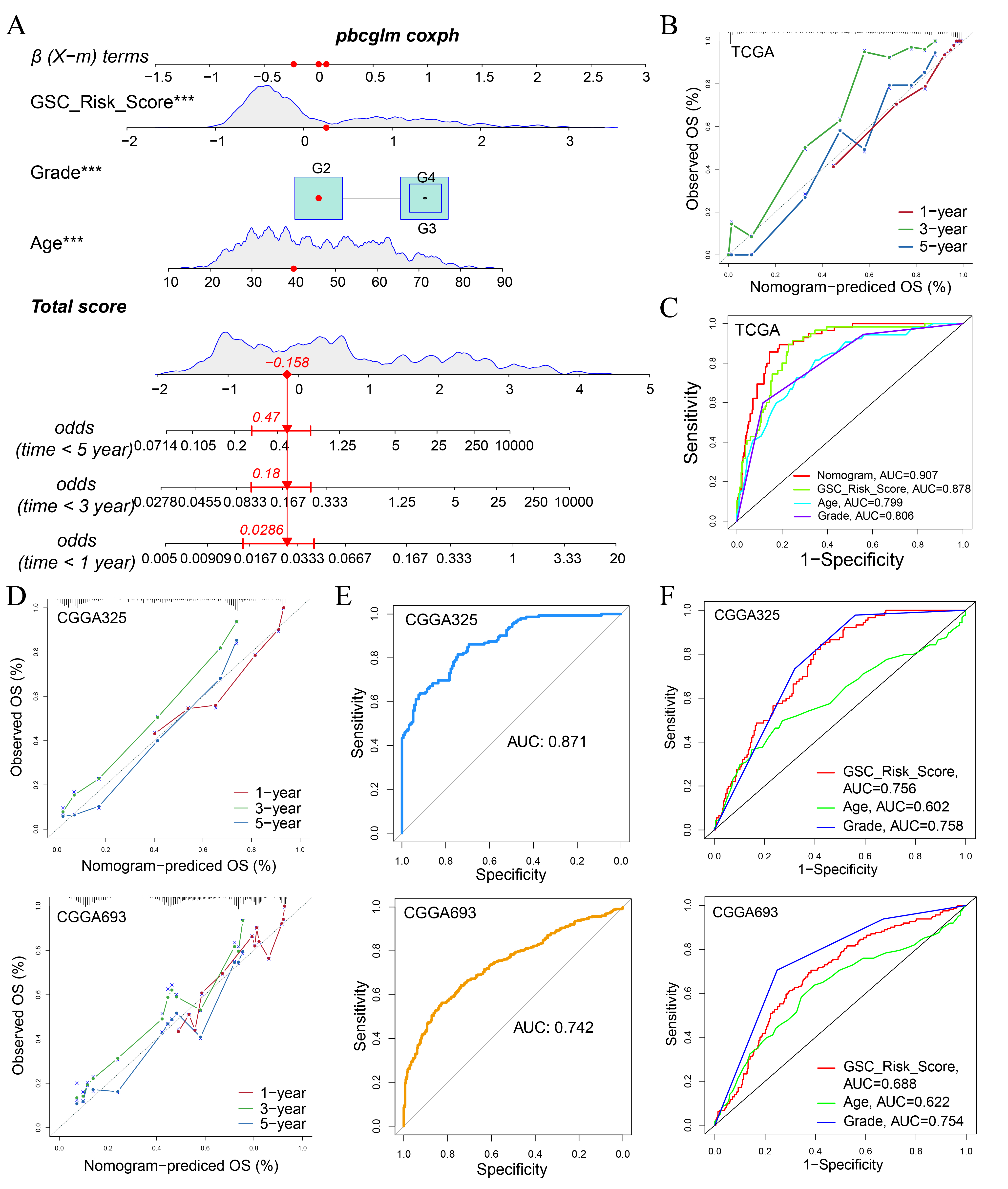

Supplement: Supplementary file 4 — Figure S4 [file CNS-28-2148-s003.jpg]

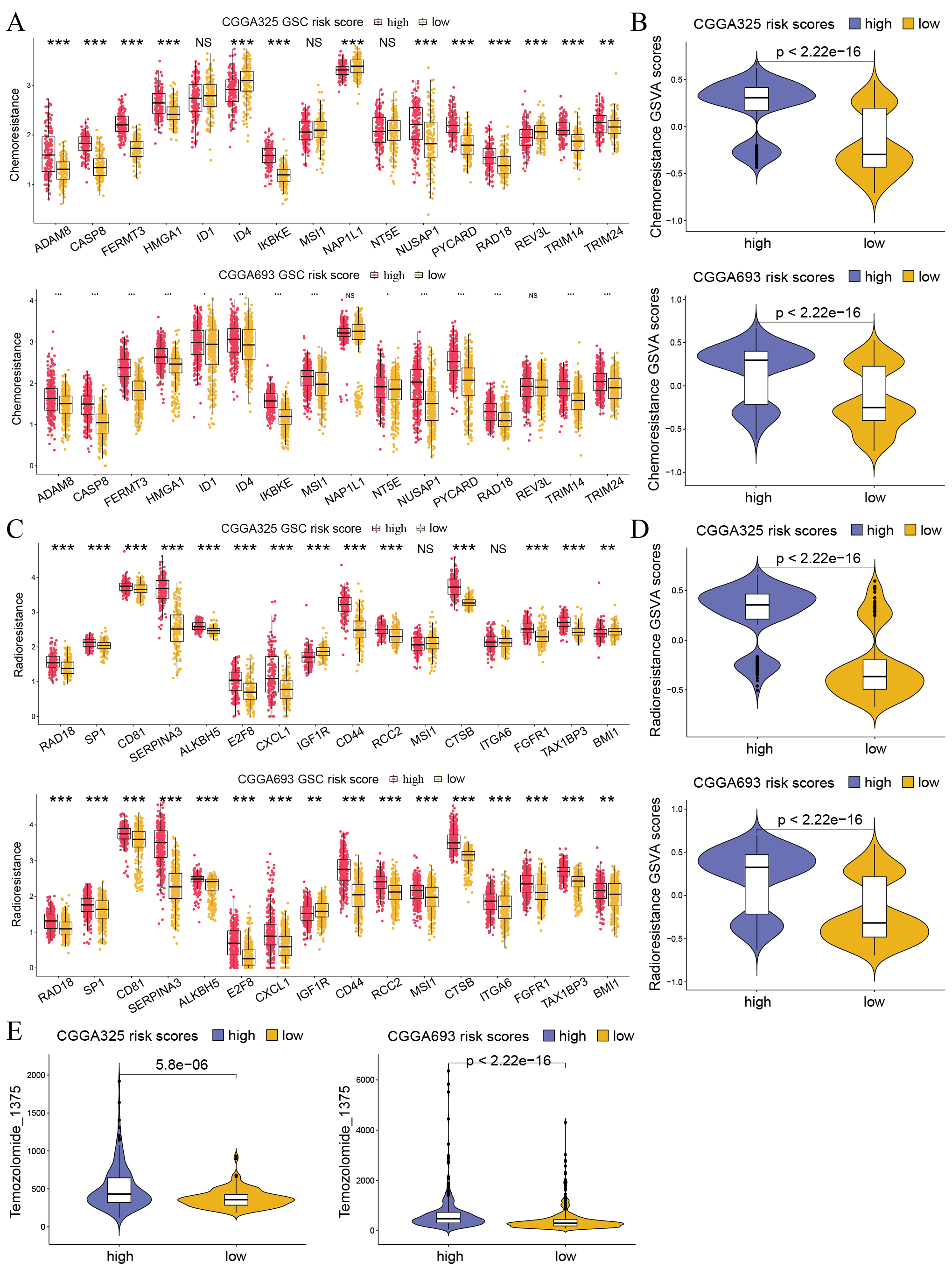

Supplement: Supplementary file 5 — Figure S5 [file CNS-28-2148-s010.jpg]

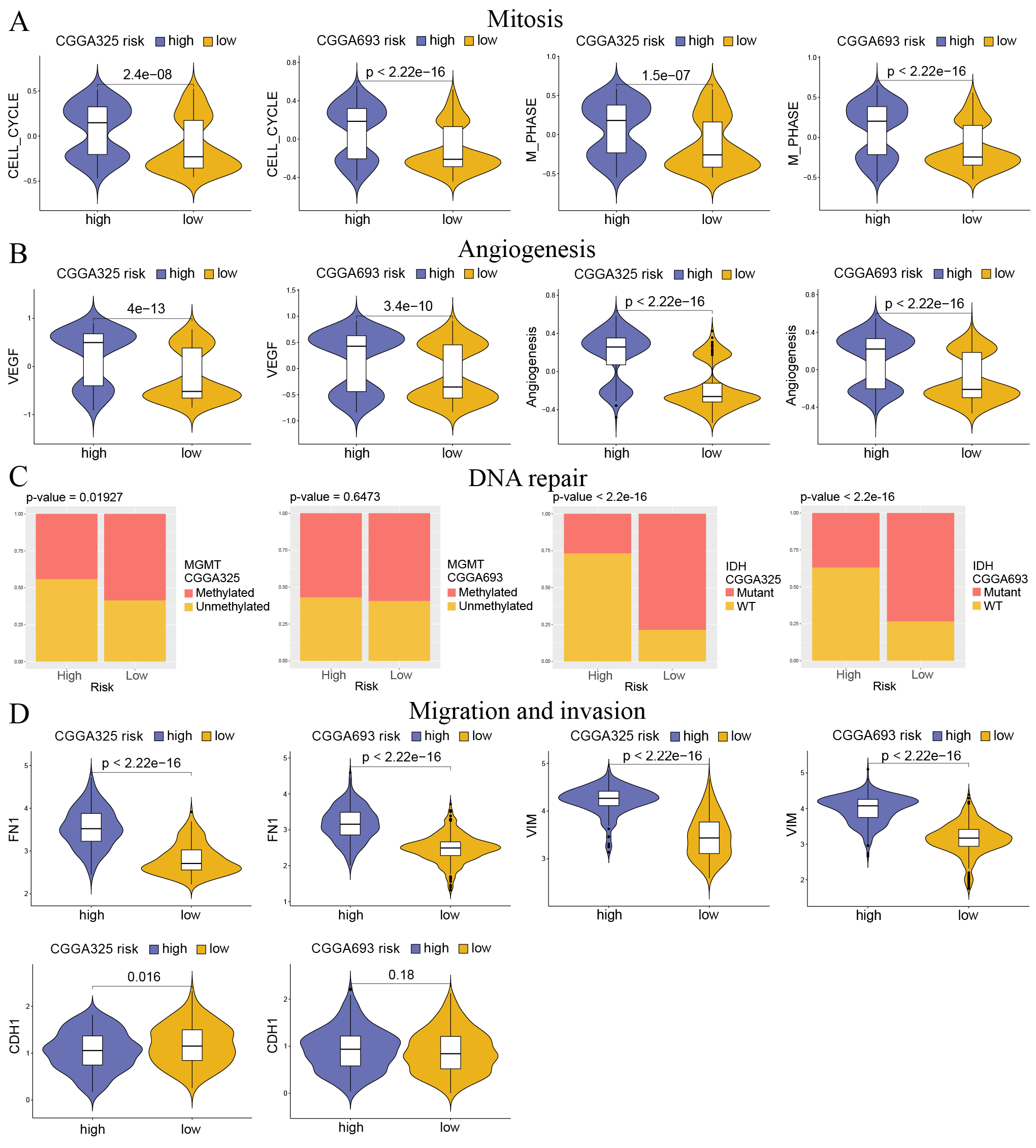

Supplement: Supplementary file 6 — Figure S6 [file CNS-28-2148-s014.jpg]

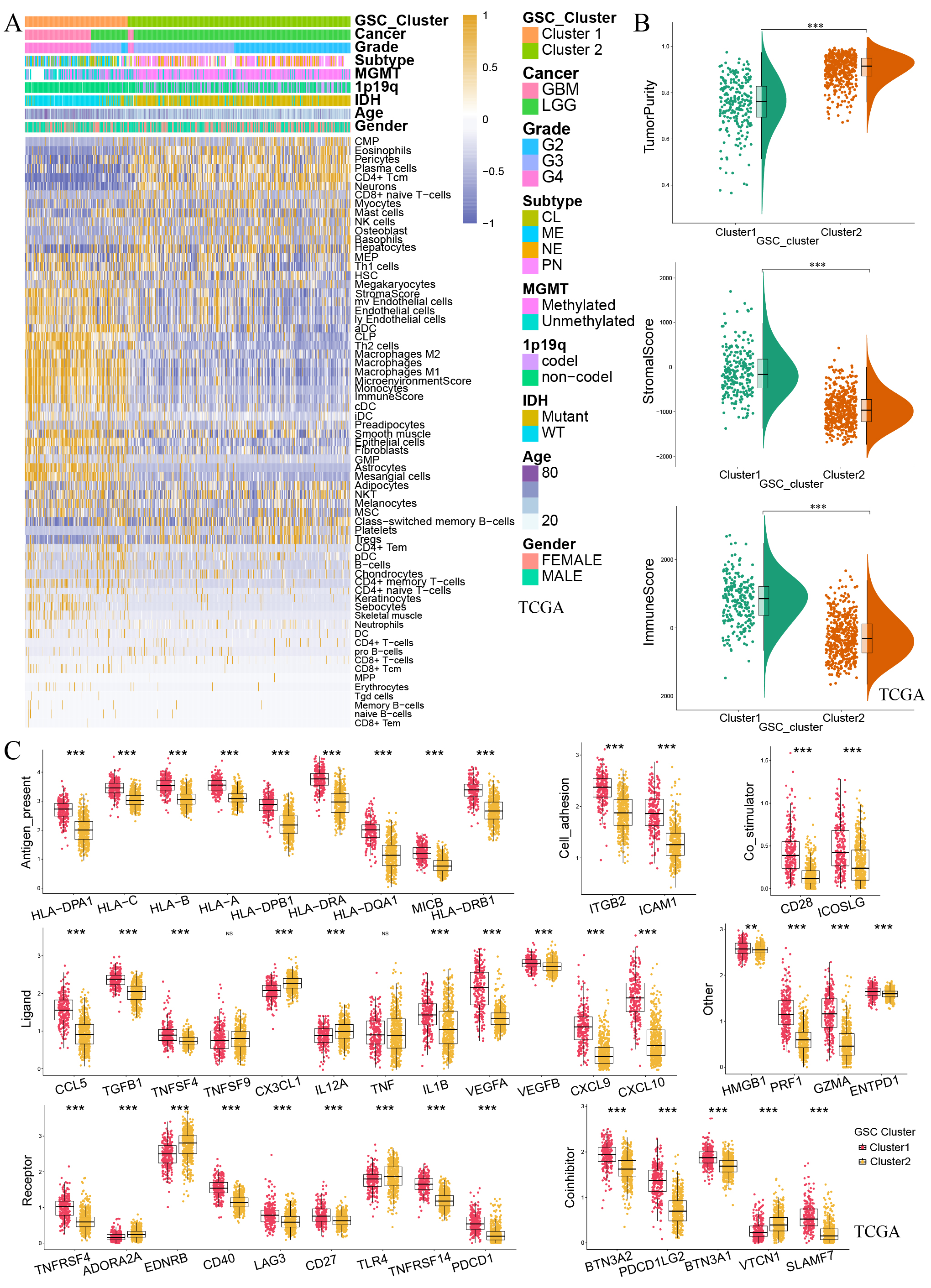

Supplement: Supplementary file 7 — Figure S7 [file CNS-28-2148-s002.jpg]

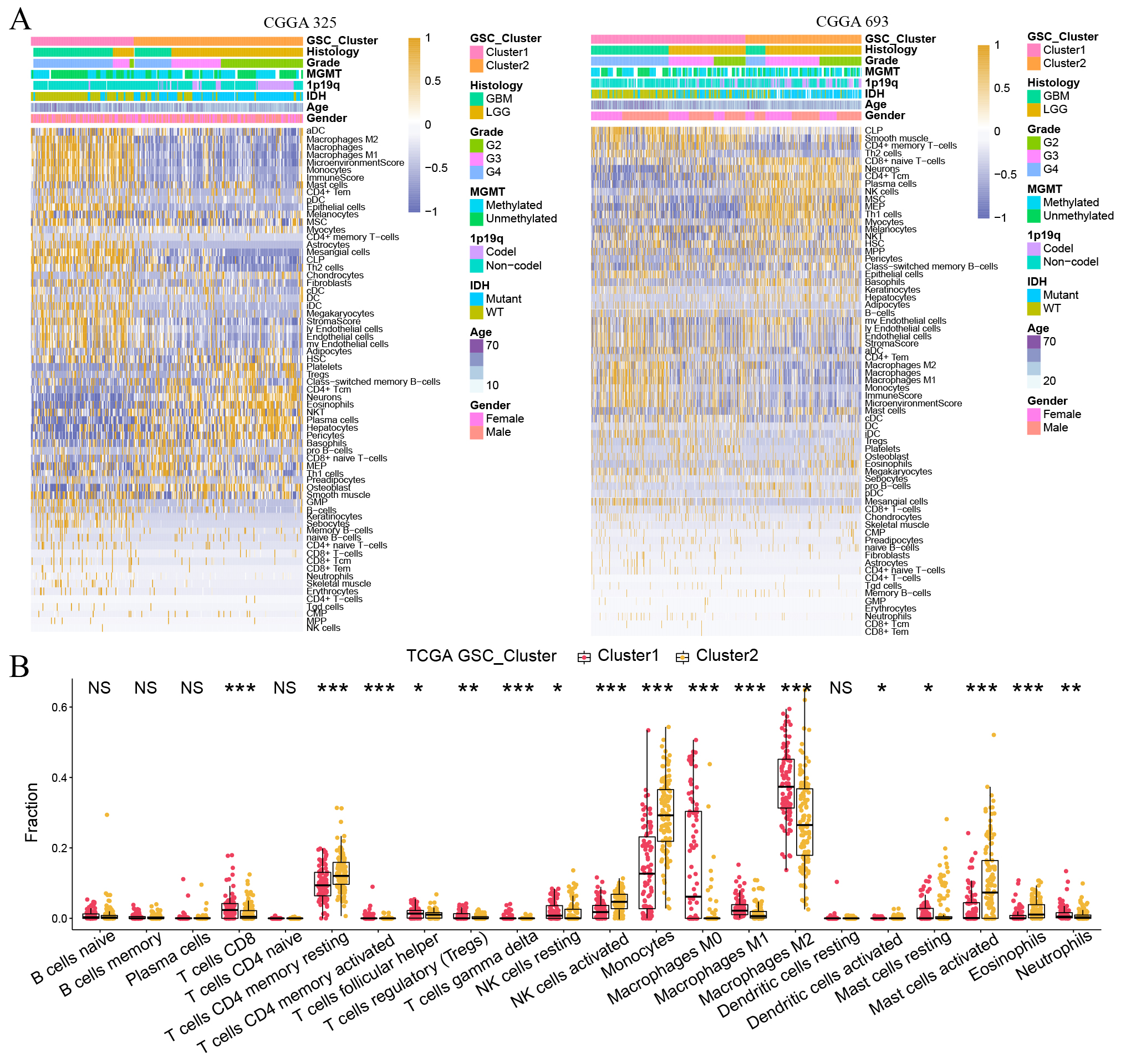

Supplement: Supplementary file 8 — Figure S8 [file CNS-28-2148-s005.jpg]

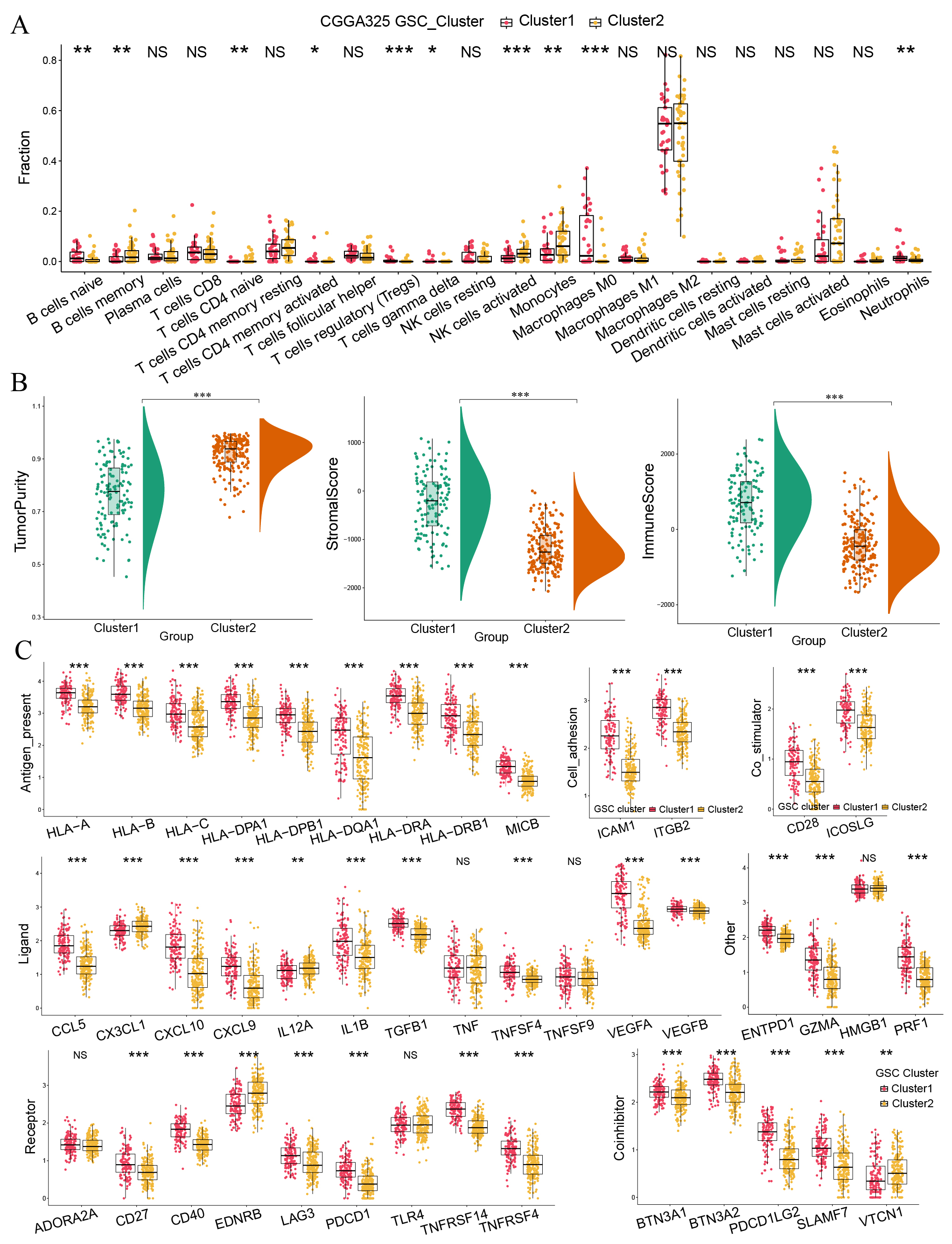

Supplement: Supplementary file 9 — Figure S9 [file CNS-28-2148-s011.jpg]

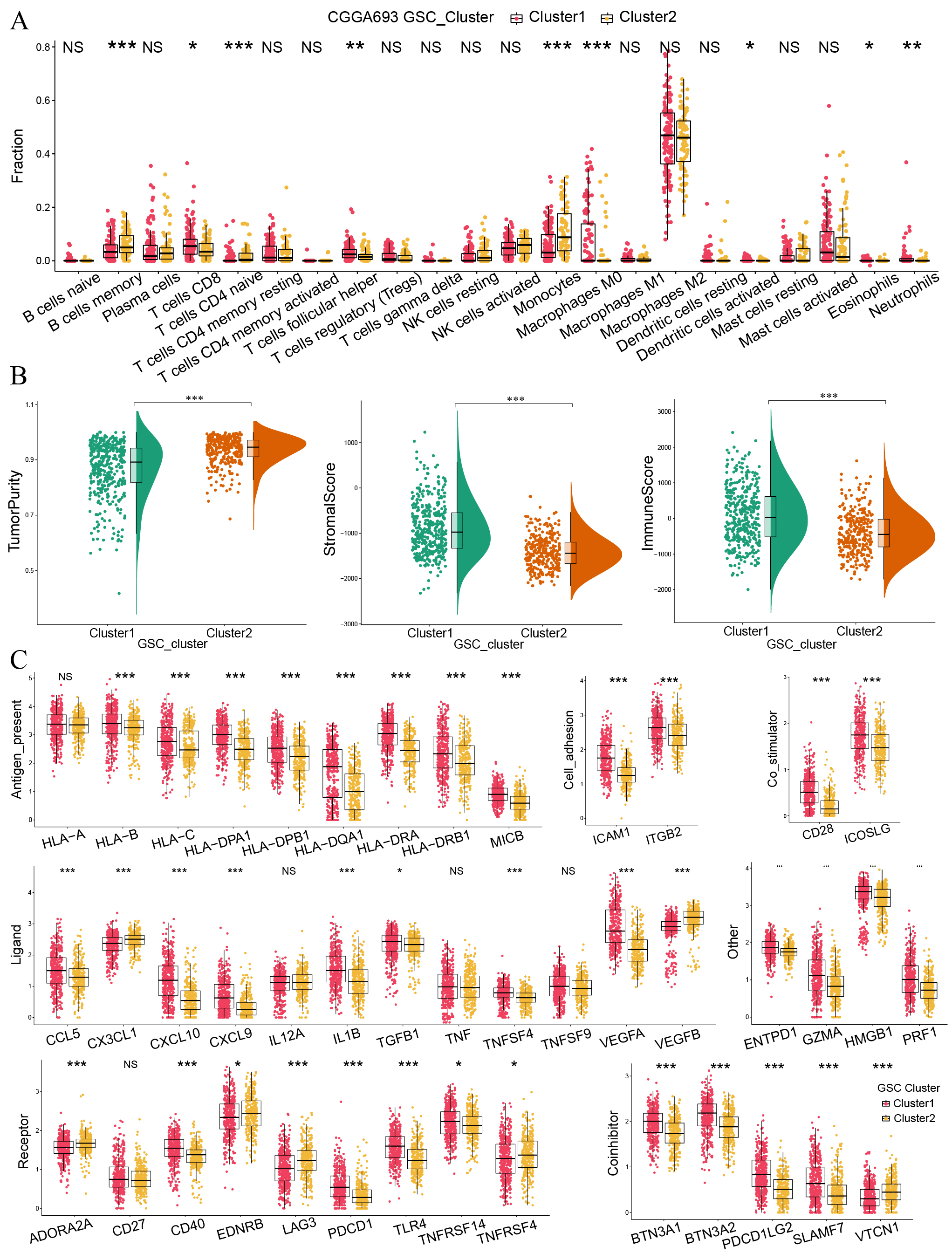

Supplement: Supplementary file 10 — Figure S10 [file CNS-28-2148-s009.jpg]

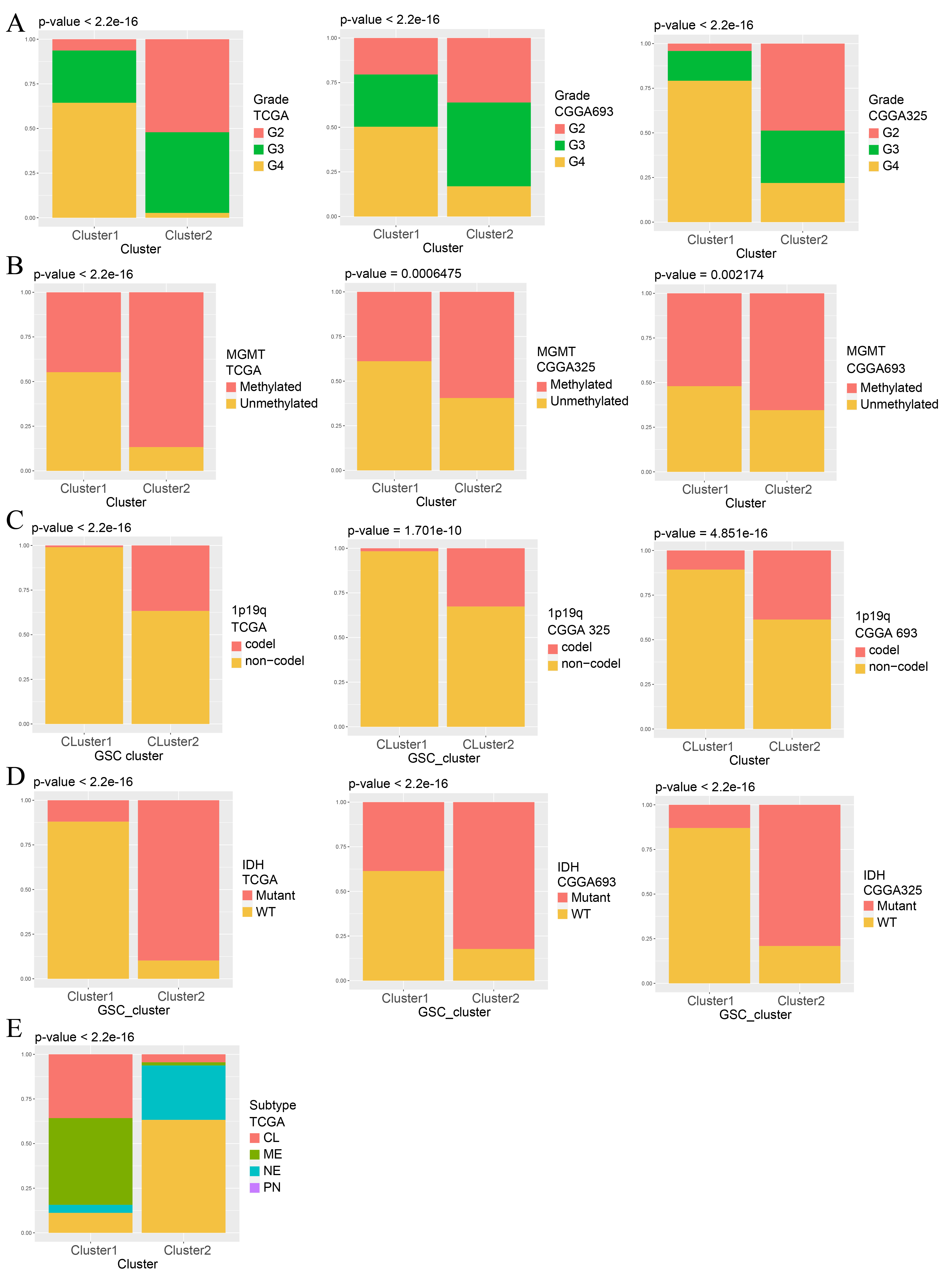

Supplement: Supplementary file 11 — Figure S11 [file CNS-28-2148-s001.jpg]

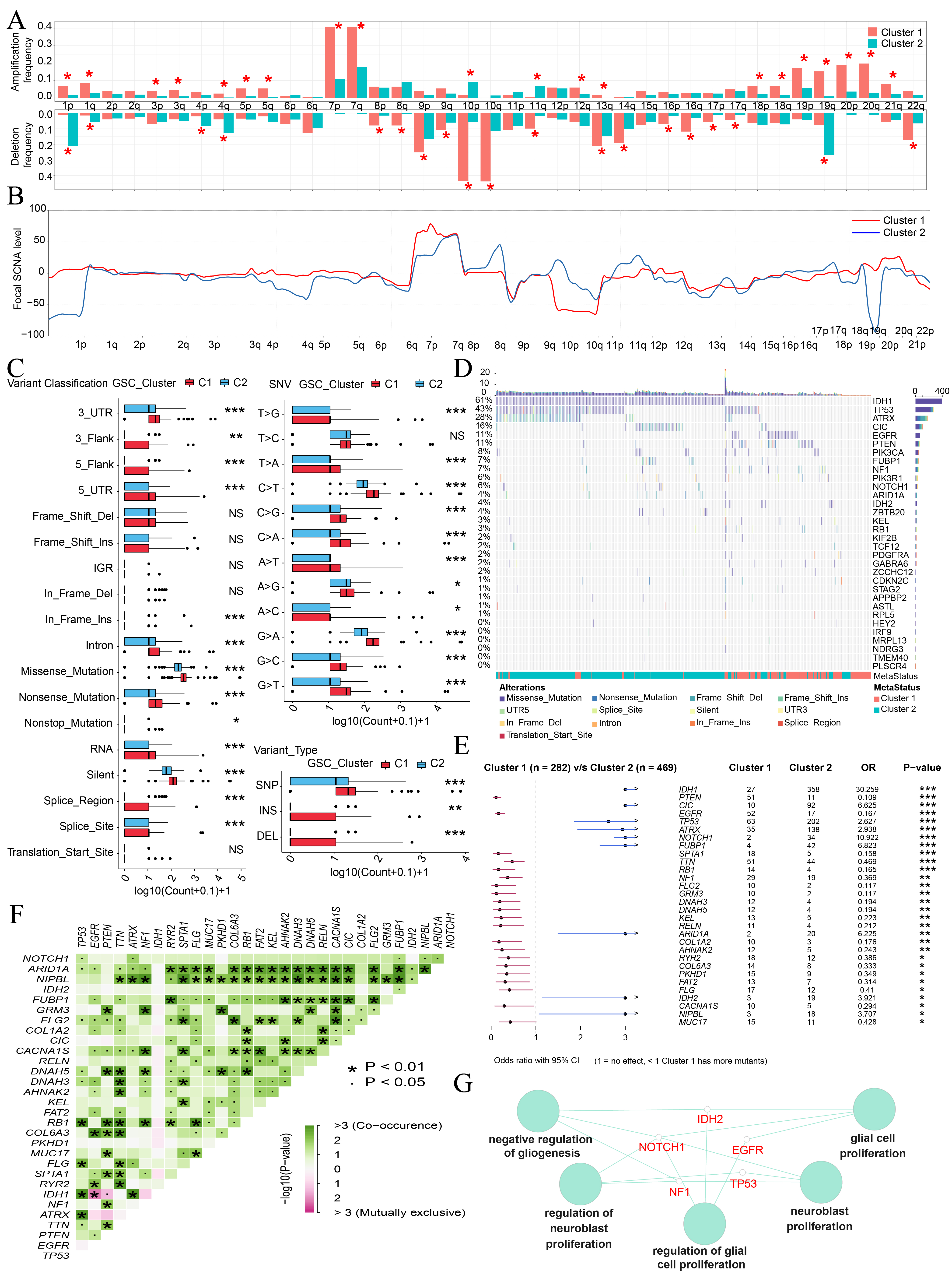

Supplement: Supplementary file 12 — Figure S12 [file CNS-28-2148-s012.jpg]

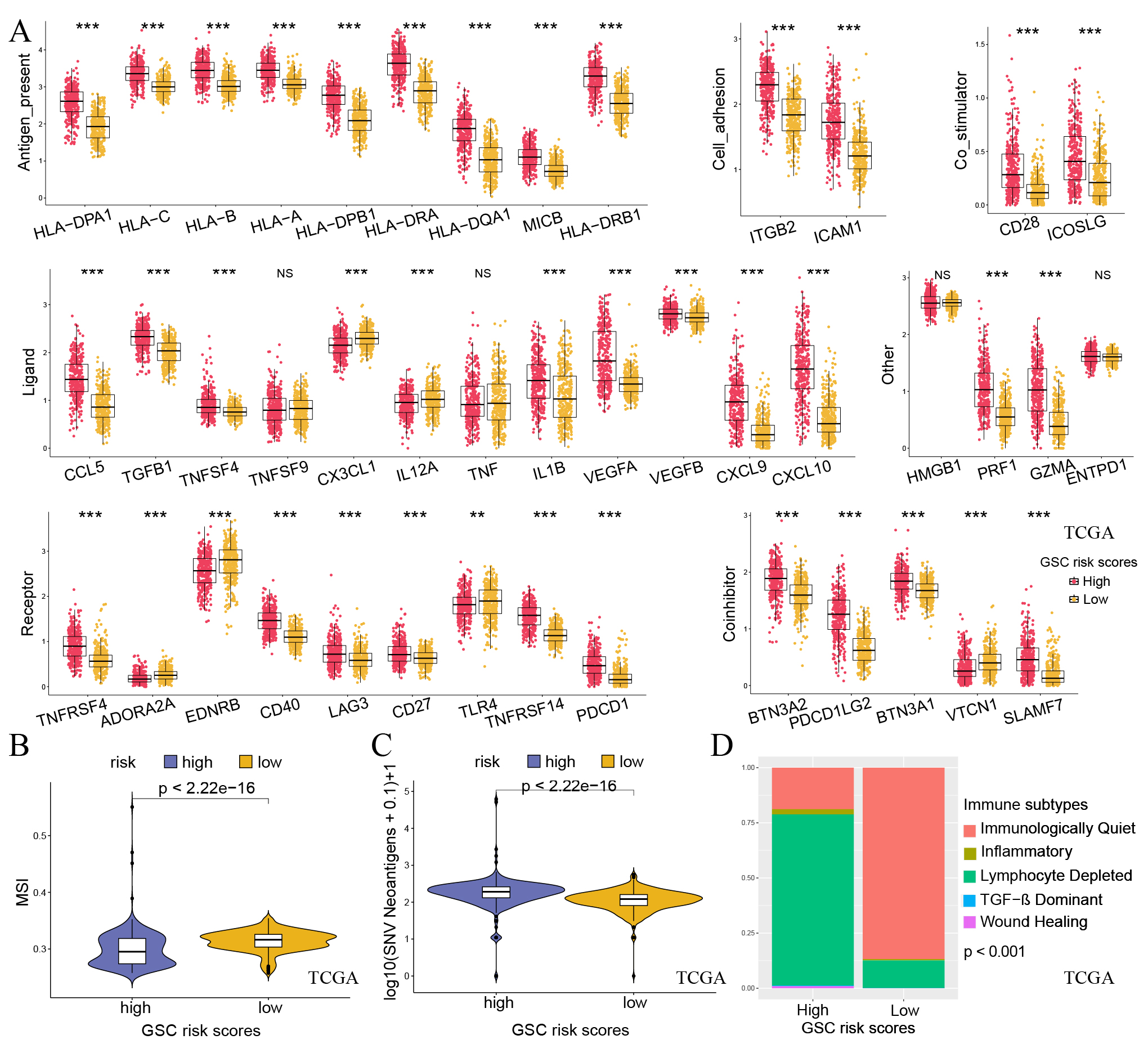

Supplement: Supplementary file 13 — Figure S13 [file CNS-28-2148-s006.jpg]

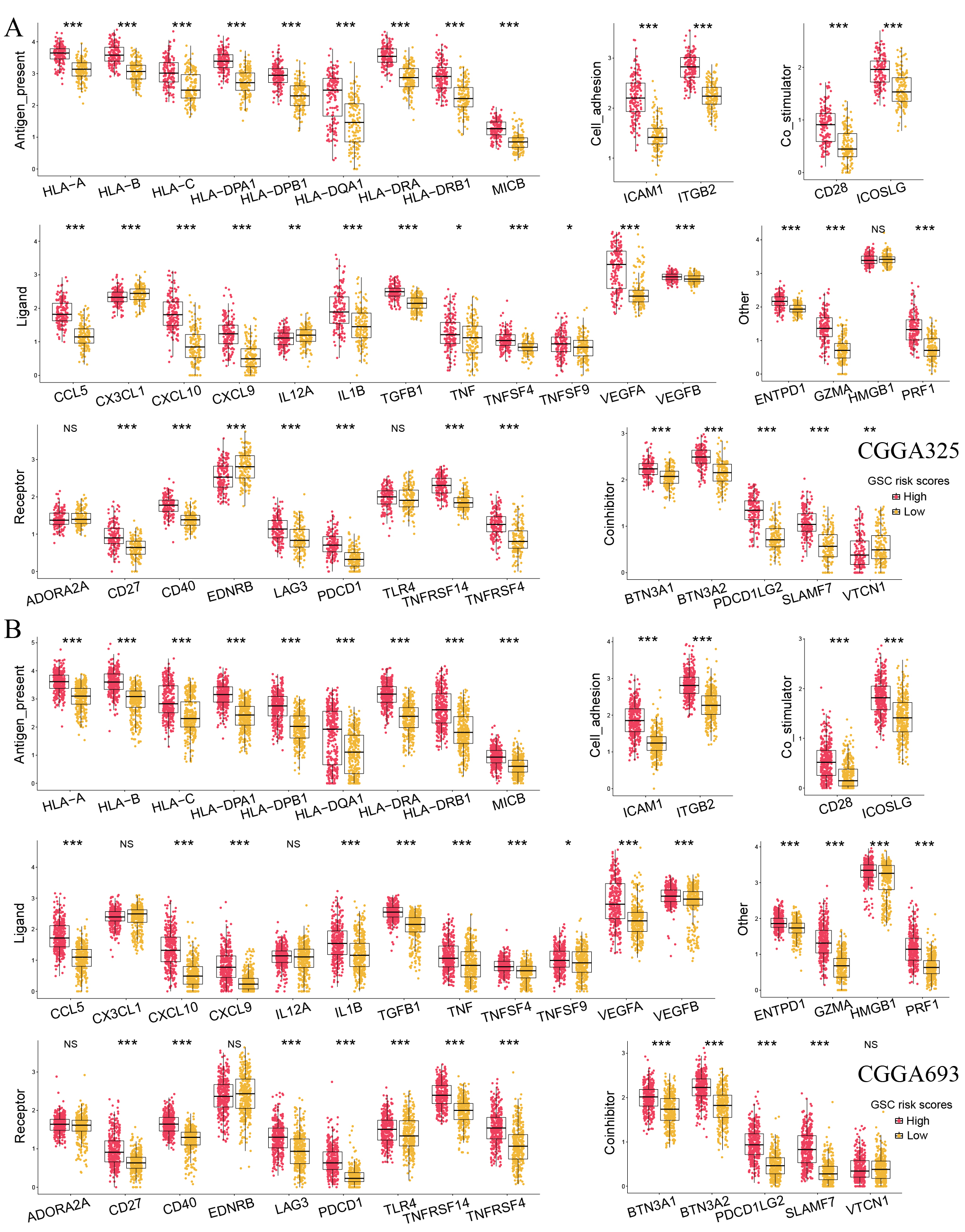

Supplement: Supplementary file 14 — Figure S14 [file CNS-28-2148-s007.jpg]
